# Contact-engineered reconfigurable two-dimensional Schottky junction field-effect transistor with low leakage currents

**DOI:** 10.1038/s41467-023-39705-w

**Published:** 2023-07-17

**Authors:** Yaoqiang Zhou, Lei Tong, Zefeng Chen, Li Tao, Yue Pang, Jian-Bin Xu

**Affiliations:** 1 Department of Electronic Engineering and Materials Science and Technology Research Center, The Chinese University of Hong Kong, Hong Kong, SAR China; 2 School of Optoelectronic Science and Engineering and Collaborative Innovation Center of Suzhou Nano Science and Technology, Soochow University, 215006 Suzhou, China; 3 Key Lab of Advanced Optoelectronic Quantum Architecture and Measurement (Ministry of Education), School of Physics, Beijing Institute of Technology, 100081 Beijing, China

**Keywords:** Electronic devices, Electronic devices

## Abstract

Two-dimensional (2D) materials have been considered promising candidates for future low power-dissipation and reconfigurable integrated circuit applications. However, 2D transistors with intrinsic ambipolar transport polarity are usually affected by large off-state leakage currents and small on/off ratios. Here, we report the realization of a reconfigurable Schottky junction field-effect transistor (SJFET) in an asymmetric van der Waals contact geometry, showing a balanced and switchable n- and p-unipolarity with the *I*_ds_ on/off ratio kept >10^6^. Meanwhile, the static leakage power consumption was suppressed to 10^−5^ nW. The SJFET worked as a reversible Schottky rectifier with an ideality factor of ~1.0 and a tuned rectifying ratio from 3 × 10^6^ to 2.5 × 10^−6^. This empowered the SJFET with a reconfigurable photovoltaic performance in which the sign of the open-circuit voltage and photo-responsivity were substantially switched. This polarity-reversible SJFET paves an alternative way to develop reconfigurable 2D devices for low-power-consumption photovoltaic logic circuits.

## Abstract (web summary)

Here, the authors report the realization of WSe_2_ Schottky junction field-effect transistors with asymmetric multi-layer graphene and WTe_2_ van der Waals contacts, enabling reconfigurable polarity, low off-state currents, near-ideal rectifying behaviour and bipolar photovoltaic response.

## Introduction

Emerging reconfigurable technologies based on two-dimensional (2D) materials are expected to empower next-generation electronic devices with lower power consumption and higher expressive capability^1–5^. Different from conventional complementary metal-oxide semiconductor (CMOS) technologies, 2D-materials-based transistors show the dopant-free polarity control that the conduction mechanism reversibly switches between n-type and p-type operation modes under gate-voltage sweeps^6–8^. These transistors are regarded as Schottky junction field-effect transistors (SJFETs), in which the carrier injection is determined by the Schottky barrier (SB) formed at the metal/semiconductor interfaces (MSIs)^9,10^. However, this ambipolar behavior renders the SJFET hard to be switched off properly^11^. Especially in downscaling of 2D SJFET, the strong electrostatic control can shrink the off-state SB, resulting in a larger leakage current and a smaller current on/off ratio, which hampers its application towards the large-scale integration and low static power consumption^12,13^.

Contact engineering as an effective modulation method has been proposed to suppress ambipolarity under gate-voltage sweeps and achieve an ultra-low off-state current in the SJFET^14–17^. The ambipolar-to-unipolar conversion of transport polarity control in 2D SJFET has been realized by introducing dual gates under the source and drain, respectively, where the injection and conduction of carriers can be individually modulated^4,18^. But the multi-gate layout in integrated circuits impeded device scaling due to gate-length limitations. The insertion of an atomically thin insulator layer such as hexagonal boron nitride or the air gap in MSI was also effective in improving the interface quality and suppressing the drain leakage^19,20^. The tunneling conduction mechanism, however, inevitably decreases the on-state current density. An easier method to build unipolar SJFET with an uncompromised on-/off-state current ratio is to use asymmetric source/drain electrodes with different work functions^21,22^. However, the strong Fermi-level pinning induced by the metal-induced gap states (MIGs) and trap-induced gap states between deposited metal/2D semiconductor interfaces usually generates an unpredictable SB height and brings the uncertainty to control the unipolarity of SJFETs^21^.

Emerging layered semimetals/metals provide a state-of-the-art approach for building the MSI to achieve ambipolar-to-unipolar conversion in van der Waals (vdWs) layered SJFETs. The bond-free integration is unaffected by lattice mismatch, or defects induced in metal deposition process to avoid trap states in the MSI^7^, making the SB height more controllable. Furthermore, semimetals with a near-zero density of states at the Fermi level have been verified to avoid MIGs and achieve the ideal MSIs^23^. Graphene as a typical Dirac semimetal, whose Fermi energy can be effectively tuned by electrostatic gating has the potential to build the reconfigurable barrier transistor^24^. Beyond graphene, the family of transition metal dichalcogenide (TMD) also furnishes a rich variety of semimetals, e.g., 1T′-MoTe_2_^25,26^, 1T′-WTe_2_^27,28^, 1T′-PtSe_2_^29^, and 2H-NbSe_2_^30^. These semimetals possess a broad range of work functions and are expected to create predictable and high-quality all vdWs Schottky junctions^31,32^.

Here, we reported on a runtime reconfigurable WSe_2_ SJFET with epitaxially-grown WTe_2_ and mechanically exfoliated multi-layer graphene (MGr) contacts, in which the WTe_2_ contact effectively suppressed the carrier injection to realize the ambipolar-to-unipolar polarity conversion controlled by the single bottom gate. Because carrier injection only allowed tunneling from the MGr contact, the WSe_2_ SJFET shows an alternative carrier polarity between n-type and p-type, by applying positive and negative source-drain voltage *V*_ds_, respectively. The SJFET under both p-type and n-type unipolarity conditions suppressed the leakage currents to 2 × 10^−10^ μA/μm, while the controllable *I*_ds_ on/off ratios with a maximum of 10^6^ retained. The static power consumption induced by the leakage off-state *I*_ds_ current was suppressed to 10^−5^ nW. The SJFET also worked as an electrically gate-tunable Schottky rectifier with a near-unity ideality factor of ~1.0 and a high rectifying ratio of 3×10^6^. The asymmetrically contacted SJFET showed a reconfigurable photovoltaic performance with the open-circuit voltage *V*_oc_ substantially tuned from 0.29 V to −0.47 V and the self-powered photoresponsivity markedly tuned from 61.7 to −12.7 mA/W. Both the negatively and positively gate-biased asymmetric photodiodes showed high filling factors with a maximum of 0.68, indicating larger shunt resistance and smaller leakage. As a facile design method, the WTe_2_/MGr contact strategy is also applicable to other 2D materials such as WTe_2_/MoS_2_ gate-tunable n-type Schottky diode, to boost 2D reconfigurable SJFETs in applications towards low- static-power-consumption and run-time reversible photovoltaic electronics.

## Results and discusssion

### The conversion from ambipolarity to reconfigurable unipolarity

**Fig. 1:**
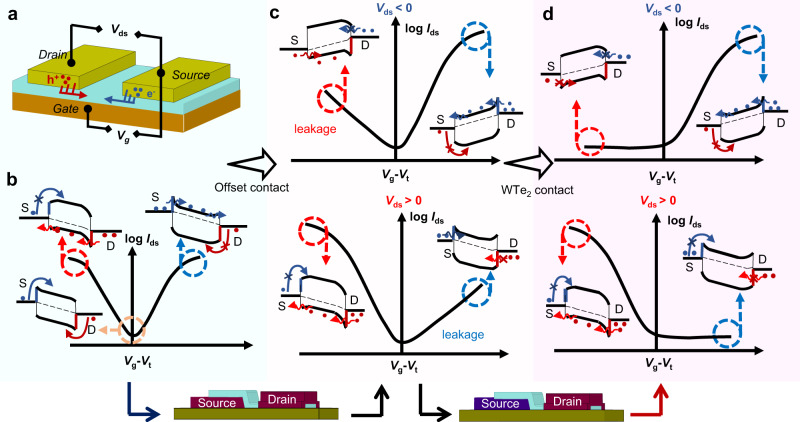
The transition from ambipolarity to unipolarity behavior of the Schottky junction field-effect transistor (SJFET). **a** Schematic of global gated SJFET with symmetric drain (D) and source (S) contacts. **b** Schematic of ambipolar transport behavior and corresponding band structure diagram. Both n- and p-branch on-state currents are attributed to the tunneling current due to the barrier reduction and thinning. TE represents thermionic emission. DT represents directing tunneling. **c** Offset contact geometry enabling p-type and n-type transport behavior and the corresponding band structure diagram. **d** Suppression of leakage currents by utilizing WTe_2_ bottom contacts. The red and blue circles represent the hole and electron, respectively. The arrows represent the carrier injection. *V*_g_ and *V*_ds_ are the gate voltage and drain–source voltage, respectively. *I*_ds_ is the drain–source current. *V*_t_ is the threshold voltage.

The ambipolarity of the SJFET is attributed to the energy level alignments and evolution at both the source and drain Schottky contacts. Figure 1a shows the schematic of the SJFET with intrinsic ambipolar transport polarity, with its band alignment evolution and the corresponding ambipolar transfer curves schematically shown in Fig. 1b. According to the general theory of the Schottky barrier based on 2D semiconductors^9^, there are two main types of carrier injection mechanisms: (1) thermionic emission (TE) when the gate voltage *V*_g_ is smaller than flat band voltage *V*_FB_, which is given by Eqs. (1) and (2):

1 $${I}_{{{{{\rm{thermal}}}}}}\,\approx \,A{T}^{2}{{\exp }}\left(\frac{q{{{{{{\rm{\varphi }}}}}}}_{{{\rm{B}}}}}{{k}_{{{\rm{B}}}}T}\right)={{AT}}^{2}\exp \left(\frac{q\left({{{{{{\rm{\varphi }}}}}}}_{{{{{{\rm{SB}}}}}}-{{{{{\rm{n}}}}}}}+{{{{{{\rm{\psi }}}}}}}_{{{{{{\rm{S}}}}}}}\right)}{{k}_{{{{{{\rm{B}}}}}}}T}\right)$$

2 $${q{\psi}}_{{{{{\rm{S}}}}}}\,\approx \,\left | \frac{{V}_{{{{{\rm{g}}}}}}-{V}_{{{{{\rm{FB}}}}}}}{{\gamma}}\right | ,\,{\gamma}\,\approx \,1+\frac{{C}_{{{{{\rm{s}}}}}}+{C}_{{{{{\rm{it}}}}}}}{{C}_{{{{{\rm{ox}}}}}}}$$

where is the φ_B_ barrier height, ψ_s_ is the surface potential, γ is the inverse band movement factor and calculated by the semiconductor capacitance *C*_S_, the interface trap capacitance *C*_it_, and the oxide capacitance *C*_ox_. (2) thermally assisted Fowler–Nordheim tunneling (FNT) and direct tunneling (DT) when the *V*_g_ exceeds the *V*_FB_, which is given by Eqs. (3) and (4):

3 $${I}_{{{{{\rm{tunnel}}}}}}=\frac{2{{{{\rm{h}}}}}}{q}{\int }_{q{{\varphi}}_{{{{{\rm{s}}}}}}}^{{{\varphi}}_{{{{{\rm{SB}}}}}-{{{{\rm{n}}}}}}}f(E){M}_{2{{{{\rm{D}}}}}}(E){T}_{{{{{\rm{WBK}}}}}}(E){{{{\rm{d}}}}}E$$

4 $${T}_{{{{{\rm{WBK}}}}}}(E)=\exp \left(-\frac{8{\pi}}{3{{{{\rm{h}}}}}}\sqrt{2{m}^{*}{({{\varphi}}_{{{{{\rm{SB}}}}}-{{{{\rm{n}}}}}}-E)}^{3}}\frac{{{{\lambda }}}_{{{{{\rm{SB}}}}}}}{q{{\psi}}_{{{{{\rm{S}}}}}}}\right)$$

where is the *f* (*E*) is the Fermi–Dirac distribution of the contact metal, *M*_2D_(*E*) is the number of 2D conduction modes in the semiconducting channel, and the *T*_WKB_ (*E*) is the SB transmission probability, and the λ_SB_ is the characteristic length. As shown in Fig. 1b, when a positive *V*_g_ > *V*_FB_ is applied, the ψ_s_ increases and the energy-band diagram is bent upwards to enable an electron tunneling current *I*_tunnel_ from source to drain, which significantly contributes to the on-state current *I*_on_ on the n-branch. Reversely, when a negative *V*_g_ < *V*_FB_ is applied, the ψ_s_ decreases, and the downward-bent band induced hole injection from the drain through direct tunneling, which contributes to the *I*_on_ on the p-branch. By setting *V*_g_ at zero, the DT current is suppressed, and only the lower TE current contributes to the off-state current due to the larger width of the contact barrier. Hence, when *V*_g_ exceeds the *V*_FB_, both n- and p-*I*_on_ are dominated by the *I*_tunnel_ through the narrowed barrier width.

To achieve the ambipolar-to-unipolar transition, we proposed an offset contact strategy to suppress the carrier injection from the source by utilizing the shielding effect of the bottom electrode to avoid electrostatically ψ_s_ shifting (Fig. 1c). Firstly, we designed an SJFET with the MGr offset contacts, as shown in Supplementary Fig. 1a, in which the source and drain were contacted at the bottom and the top surfaces of the WSe_2_ channel, respectively. Supplementary Fig. 1b–c show the transfer curves of the WSe_2_ FET at various *V*_ds_ with the offset contacts. When *V*_ds_ < 0, the WSe_2_ transistor showed a p-type transport polarity with an on-state *I*_ds_ of 0.18 μA/μm. Reversely, the WSe_2_ transistor showed an n-type polarity when *V*_ds_ > 0. The output curves at negative and positive *V*_g_ also indicated that the back-to-back junctions were asymmetrically modulated, as shown in Supplementary Fig. 1d. However, the carrier injection from the source side was only suppressed slightly, as shown in Supplementary Fig. 1e–g, which induced a higher off-state *I*_ds_ of 0.4 × 10^−2^ μA/μm and a lower on/off ratio (~10) for the MGr-contacted WSe_2_ SJFET. This weak ambipolar-to-unipolar transition was hard to be simply explained by the air-gap-induced barrier widening^20^. The contact interaction between the WSe_2_ and the bottom contact also played an important role to repress the leakage current, which will be discussed in Part III. According to our previous report based on the MGr/MoS_2_/WTe_2_ vertical junction^33^, WTe_2_ possessing an appropriate work function and weak interlayer interaction with WSe_2_ is expected to enlarge the tunneling width of SB at WTe_2_/WSe_2_ interface. Therefore, we can optimize the bottom contact material by utilizing WTe_2_ to suppress the leakage currents and fulfill the ambipolar-to-unipolar transition by only allowing carrier injection from the top contact side, as shown in Fig. 1d.

### Preparation and electrical characterization of WTe_2_ contacts

**Fig. 2:**
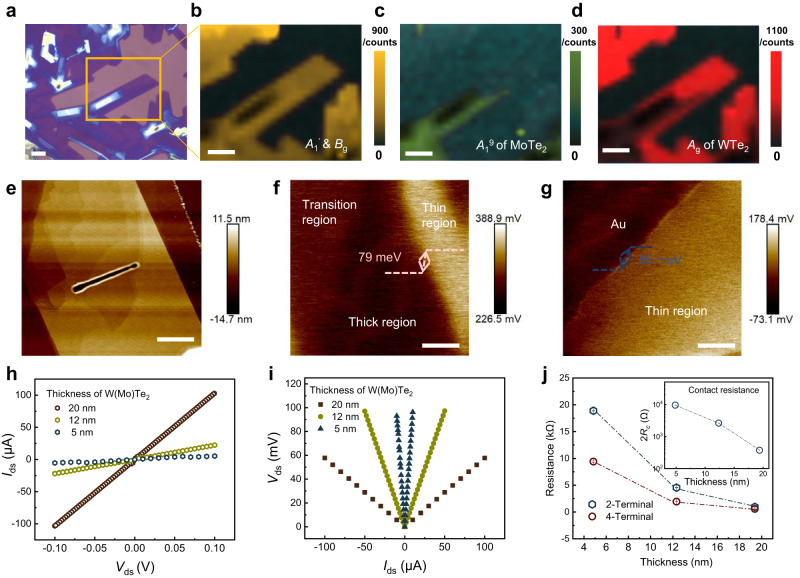
Growth and characterization of WTe_2_ bottom electrode. **a** Optical image of the epitaxially grown WTe_2_. Scale bar: 20 μm. **b**–**d** Raman intensity mapping images with the characteristic peaks corresponding to *A*_1_^7^, *A*_1_^9^ modes of WTe_2_, and *B*_g_, *A*_g_ mode of MoTe_2_. Scale bar: 20 μm. **e** Morphology of epitaxially-grown WTe_2_ measured by AFM. Scale bar: 4 μm. **f** Potential image of epitaxially-grown WTe_2_ measured by Kelvin probe force microscopy. Scale bar: 4 μm. **g** Potential image of Au and CVD-grown WTe_2_. Scale bar: 2 μm. **h** *I–V* curves of W(Mo)Te_2_ with various thicknesses measured by the 2-terminal method. **i** *I–V* curves of W(Mo)Te_2_ with various thicknesses measured by the 4-terminal method. **j** 2-terminal resistance (*R*_2T_) and 4-terminal resistance (*R*_4T_) of W(Mo)Te_2_ with different thicknesses. The standard deviations were used as error bars. The inset shows the contact resistance 2*R*_contact_ = *R*_2T_–*R*_4T_ versus the thicknesses of W(Mo)Te_2_.

To obtain a high-quality WTe_2_ bottom electrode, we proposed a one-step epitaxial growth method to prepare large-size WTe_2_. Figure 2a shows the photograph of epitaxially-grown WTe_2_ in which thicker MoTe_2_ flakes were synthesized first and worked as growth seeds to provide nucleation sites and reduce the nucleation energy barrier of WTe_2_. Compared to direct WTe_2_ growth, the epitaxial growth of WTe_2_ showed a large-size and high-quality surface and clear W(Mo)Te_2_ interface, advantageously reducing the unexpected Fermi level pinning and controlling the contact barrier precisely. More information about the sample growth and characterization is in the Methods section and Supplementary Note 1. Figure 2b–d show the Raman intensity mapping indicating the heterostructure properties and excellent uniformity of MoTe_2_ and epitaxial WTe_2_. The surface quality of the WTe_2_ was probed by atomic force microscope (AFM), as shown in Fig. 2e. Both the thin WTe_2_ at the edge and the thick MoTe_2_ possessed smooth surfaces, which were expected to work as the vdWs Schottky contact with a clean and desired interface.

As a promising candidate to build the semimetal-semiconductor junction, the potential and resistance of epitaxially-grown WTe_2_ were further investigated. Figure 2f shows the surface-potential image of WTe_2_ measured by Kelvin probe force microscopy (KPFM). The edge WTe_2_ possessed a higher surface potential compared to the thick MoTe_2_ region and the difference in potential between MoTe_2_ and WTe_2_ was 79 meV. The work function (WF) of WTe_2_ was ~5.17 eV by using Au film (WF_Au_ = 5.1 eV) as the reference (Fig. 2g). Figure 2h and i show the *I*_d_–*V*_d_ curves of W(Mo)Te_2_ with various thicknesses characterized by 2-terminal and 4-terminal methods, respectively. The measurements are detailed in Supplementary Fig. 5. Since the 4-terminal resistance (*R*_4T_) eliminated the contact resistance, it was used to assess the intrinsic electrical resistance of the WTe_2_ (The details were discussed in Supplementary Note 2). The WTe_2_ with a thickness *t* of ~5 nm showed the highest resistance of about *R*_4T_ = 9.41 kΩ. For the sample with thickness between *t* = 5–20 nm, the resistance decreased to 1.95 kΩ and the bulk sample (*t* > 20 nm) possessed the lowest conductivity of 0.58 kΩ (Fig. 2i). Compared to the *R*_4T_, the 2-terminal resistance (*R*_2T_) showed a more pronounced change with thickness, indicating the contact resistance 2*R*_contact_ = *R*_2T_–*R*_4T_ between the transferred Au film and W(Mo)Te_2_ increased with the decreased thickness, as shown in the inset of Fig. 2j.

Further, the current density (*V*_ds_ = 0.1 V) of WTe_2_ devices at different temperatures was measured by the 2-terminal method (Supplementary Figs. 6–7). The current density of WTe_2_ with a small thickness of ~5 nm was positively correlated with the temperature. As the thickness increased, the WTe_2_ exhibited a weak temperature dependence. However, the WTe_2_ with a thickness larger than 20 nm showed a negative temperature coefficient of current density, as shown in Supplementary Fig. 7b–d. This metal-semiconductor transition was consistent with exfoliated W(Mo)Te_2_ flakes in previous reports^34^, which may be attributed to contact resistance and the surface absorption of the hydroxyl group in ambient conditions. The transfer curves of WTe_2_ with varied thicknesses also support this transition (Supplementary Fig. 7e–g). The bulk sample exhibited a constant source-drain current *I*_ds_ when the gate-voltage *V*_g_ swept, but the *I*_ds_ measured in the thin WTe_2_ were modulated by gate-voltage steadily, showing a weak p-type characteristic, especially in the low-temperature range.

### Carrier injection capability comparison between MGr and WTe_2_

**Fig. 3:**
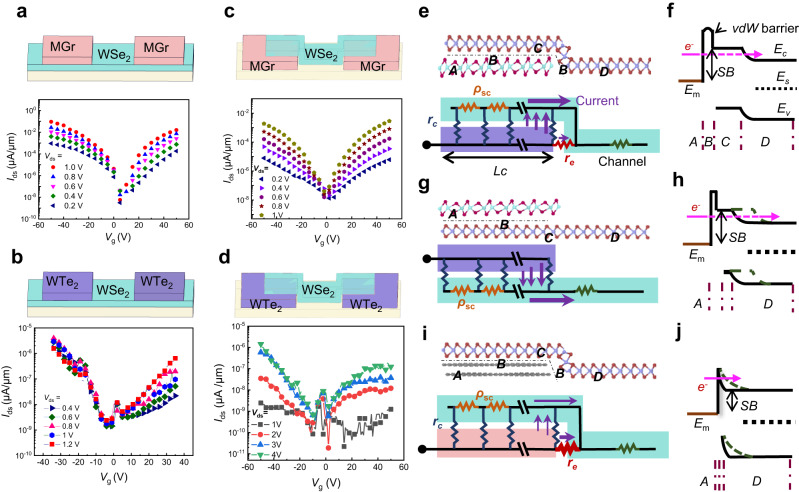
Carrier injection capability of the bottom MGr and WTe_2_ contacts. **a** Schematic and the transfer curves of MGr top-contacted FET. **b** Schematic and the transfer curves of WTe_2_ top-contacted FET. **c** Schematic of and the transfer curves MGr bottom-contacted FET. **d** Schematic and the transfer curves of WTe_2_ bottom-contacted FET. **e**, **f** Schematic of bottom-contacted WTe_2_-WSe_2_ and the network of contact resistances, and the corresponding schematic of the energy-band diagram. *E*_c_, *E*_v_, and *E*_s_ represent the energies of the conduction band, valence band and Fermi level in 2D semiconductor, respectively. *E*_m_ is the work function of the contact metal. SB represents the Schottky barrier. **g**, **h** Schematic of top-contacted WTe_2_-WSe_2_ and network of contact resistances, and the corresponding schematic of the energy-band diagram. **i**, **j** Schematic of MGr-WSe_2_ and network of contact resistances, and the corresponding schematic of energy-band diagram. The dashed line in the energy-band diagram indicates the band evolution induced by *V*_g_. Brown spheres: selenium atoms. Rose red spheres: tellurium atoms. Green and purple spheres: tungsten atoms. Gray spheres: carbon atoms. Regions A-D represent the metal contact, the interface gap, the contacted semiconductor, and the channel semiconductor region, respectively. *ρ*sc is the sheet resistance of the semiconductor overlapped with the contact, *r*_c_ and *r*_e_ are the specific resistivities of the contact gap and edge, respectively, *L*_C_ is the contact length.

To compare the carrier injection capability of different contact materials and geometries, we built the WSe_2_-SJFET using symmetric top and bottom contacts with the exfoliated MGr and epitaxially grown WTe_2_. All devices were fabricated by the dry-transfer method to avoid the formation of defect-induced states. Figure 3a, b show the schematics and transfer curves of the top-contacted device at various *V*_ds_. Compared to the FET with top WTe_2_ contacts, the FET with MGr top electrodes showed symmetric transfer characteristics with higher currents, indicating the MGr possessed higher carrier injection efficiency for both electrons and holes, which was the reason why the MGr used as the top contact. We also measured the transfer curves of the FET with other bulk or layered metal contacts, but most of these contacts showed an asymmetric carrier injection efficiency and resulted in a stronger p-branch in the *I*_ds_–*V*_g_ curves, as shown in Supplementary Fig. 8. Figure 3c, d show the transfer curves of the bottom-contacted FET at varied *V*_ds_. The FET with MGr bottom contacts still showed a high and symmetric on-state *I*_ds_. Reversely, *I*_on_ of the WTe_2_-contacted WSe_2_ device was only ~10^−9^ μA/μm at *V*_ds_ = 1 V, which was six orders of magnitude lower than that of the MGr-contacted FET, the same behavior as shown in the output characteristics (Supplementary Fig. 9b, c). The results suggested that the WTe_2_ bottom contacts exhibited a lower carrier injection efficiency due to the self-shielding effect of bottom contact^14^, weak interfacial interaction, and vdWs*-*gap-induced tunneling barrier at the WTe_2_/WSe_2_ interface.

To explain the contact-geometry-induced suppression of carrier injection, we show the schematic of the current flow pathways in a typical MS surface contact geometry in Fig. 3e−i, where the current flow from metal (A) to channel (D) passes through two regions including the vdWs gap at MS interface (B) and the WSe_2_ overlapped with metal (C). We further reduce the surface contact region of WSe_2_ FETs into a resistor network under the diffusive approximation, and the contact *R*_C_ is expressed in the transmission line model^35^:

5 $${R}_{{{{{\rm{c}}}}}}=\sqrt{{{\rho}}_{{{{{\rm{sc}}}}}}{r}_{{{{{\rm{c}}}}}}}\coth ({L}_{{{{{\rm{c}}}}}}/\sqrt{{{\rho}}_{{{{{\rm{sc}}}}}}{r}_{{{{{\rm{c}}}}}}})$$

where ρ_SC_ is the sheet resistance of the 2D semiconductor beneath the contact, *r*_c_ is the specific resistivity of the MS interface, *L*_*c*_ is the contact length, respectively. For the top contact geometry, modulated by the global bottom-gate, ρ_SC_ was decreased as the amplitude of *V*_g_ increased, which reduced the *R*_C_ and improved the on-state currents. However, for the bottom contact geometry, ρ_SC_ was hardly tuned by the bottom gate due to the shielding effect of the electrode, resulting in a large contact resistance and a poor on-state current density. The shielding effect of the bottom electrode was also verified by simulation using the COMSOL Multiphysics package, as shown in Supplementary Fig. 10, in which both the electric field and carrier density of the WSe_2_ atop the bottom contact were hardly to be modulated by *V*_g_. Besides, the vdWs gap between the channel and the vertical side wall of the bottom contact (Supplementary Fig. 10b–o also led to the large and nonadjustable contact resistance due to the large interface resistance *r*_c_^36^, which was discussed in the previous report^20^. The same tendency could be derived from the schematic energy-band diagrams of the MS structures. As shown in Fig. 3h, the width of the n-type (p-type) Schottky barrier was narrowed as the *V*_g_ increased (decreased) in the WTe_2_ top contact geometry. In contrast, it is difficult to be modulated in the WTe_2_ bottom contact geometry (Fig. 3f).

Apart from the contact geometry, the contact materials were also important. Supplementary Fig. 11 shows the potential difference at the WSe_2_/WTe_2_, and WSe_2_/MGr interfaces measured by KPFM. Compared to the potential difference between WSe_2_ and MGr, there existed a smaller potential difference of 37 meV between WTe_2_ and WSe_2_, indicating that their WFs were horizontally aligned; therefore, WTe_2_ had a small charge transfer doping to the WSe_2_ and avoided the ψ_s_ shift of the contacted WSe_2_. Meanwhile, for the MGr bottom contact, the electrical contact was dominated by the edge interface of the MGr electrode (Fig. 3j), which was thinner than that of WTe_2_ contacts and difficult to suppress the tunneling injecting current. To verify that, the flat-band barrier heights (SHB) of the MGr bottom contact are calculated by 2D thermionic emission mode, as shown in Eq. (6)^9^:

6 $${I}_{{{{{\rm{ds}}}}}}=\left[A{A}^{*}{{{{{\rm{T}}}}}}^{1.5}\exp \left(-\frac{q{{\Phi}}_{{{{{\rm{B}}}}}}}{{k}_{{{{{\rm{B}}}}}}T}\right)\right]\left[\exp \left(\frac{q{V}_{{{{{\rm{ds}}}}}}}{{k}_{{{{{\rm{B}}}}}}T}-1\right)\right]$$

where *A* is the junction area and A* is the effective Richardson–Boltzmann constant. The obtained Φ_B_ as a function of *V*_g_ is shown in Supplementary Fig. 9, which indicated that the *I*_on_ of both p- and n-branch was based on the tunneling mechanism due to the gate-thinned barrier.

The few-layered WTe_2_ also showed a weak interfacial interaction with the orbital overlapping to WSe_2_, compared to the Au film which possessed a similar WF to WTe_2_. We measured the potential difference of WSe_2_ on Au and WTe_2_ substrate using the WSe_2_ on SiO_2_ wafer as the reference (Supplementary Fig. 12). The results showed a positive potential difference (~300 meV) between WSe_2_ on Au film and the WSe_2_ on WTe_2_ flake, indicating an unexpectedly strong doping effect of Au film due to the interfacial state, such as metal-induced gap states (MIGS), defect states, and the interface dipoles^37^. Meanwhile, we also compared the intensity and shape-variation of the Raman characteristic peaks of WSe_2_ on Au and WTe_2_ flake (Supplementary Fig. 13). For out-of-plane vibrational *A*_1g_ mode affected by the electrostatic environment change, its full width at half maximum (FWHM) was enlarged as WSe_2_ overlapped on Au film compared that of WSe_2_ overlapped on WTe_2_ (Supplementary Fig. 13c–f), indicating the strong charger transfer doping effect on Au film^38,39^. The detailed comparison is discussed in Supplementary Note 3.

### Reconfigurable unipolar WSe_2_ SJFET with asymmetric contact

**Fig. 4:**
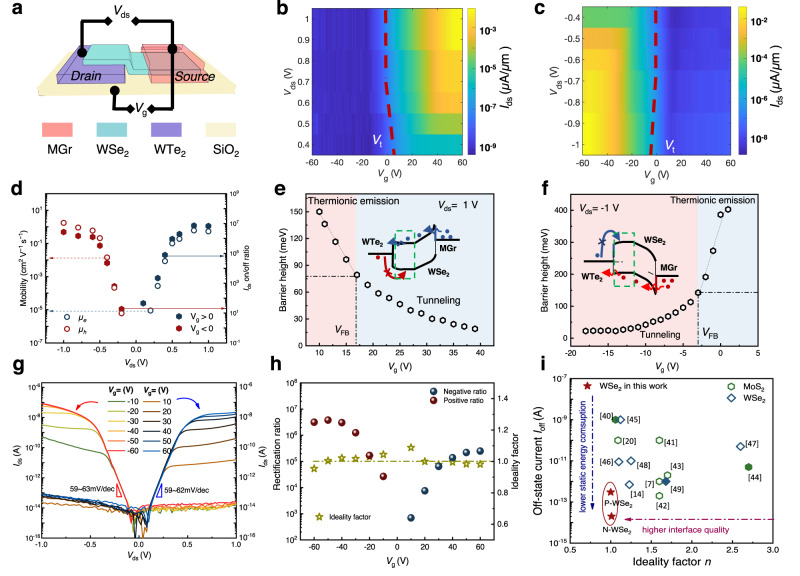
Reconfigurable WSe_2_ SJFET with asymmetric MGr/WTe_2_ contacts. **a** Schematic of asymmetrically contacted WSe_2_ SJFET. **b** Pseudo-color transfer plots of the device at *V*_ds_ > 0 showed the n-type polarity. **c** Pseudo-color transfer plots of the device at *V*_ds_ < 0 showed the p-type polarity. The red dash lines indicated the threshold voltage *V*_t_. **d** *V*_ds_-dependent effective field-effect mobility and the *I*_ds_ on/off ratios. All field-effect mobilities were extracted from the linear regimes. **e**, **f** Barrier heights of the device at *V*_ds_ = 1 V and *V*_ds_ = −1 *V*. The Schottky barrier height is extracted under a flat-band gate voltage (*V*_FB_) condition, which was responsible for the start of deviations from the linear behavior. **g** Output characteristics of the device at varied gate voltages. **h** Gate-dependent rectifying ratios of the device. The gray-green dashed line represents the mean value of the ideality factors. **i** Comparison of ideality factor *n* and off-state current of MoS_2_^7,21,41–45^ and WSe_2_^14,46–50^ SJFET in previous reports.

To suppress the ambipolar behavior while the high on-state performance retained, we fabricated a WSe_2_ SJFET with bottom-contacted WTe_2_ and top-contacted MGr electrode, as the drain and source contacts, respectively, as shown in Fig. 4a. The optical images of the devices are shown in Supplementary Fig. 9a and the thicknesses of the MGr, WSe_2_, and WTe_2_ were 13.2 nm, 6.2 nm, and 11 nm, respectively. The transistor characteristics were dominated by both source-drain polarity and control. When *V*_ds_ was positively biased, as shown in Fig. 4b, the WSe_2_ SJFET showed an n-type characteristic, and the on-state current (*I*_on_) increased to 6 × 10^−3^ μA/μm as *V*_ds_ increased to 1 V. Meanwhile, the off-state current was suppressed to ~10^−10^ μA/ μm at *V*_ds_ = 1 V and *V*_g_ = −60 V, hence, a maximum on/off ratio higher than 10^6^ was achieved.

When *V*_ds_ was negatively biased shown in Fig. 4c, the WSe_2_ FET displayed a p-type characteristic with *I*_on_ of 1.1 × 10^−2^ μA/μm at *V*_ds_ = −1V. Similar to the n-type one, *V*_t_ increased as the amplitude of *V*_ds _ decreased, but the leakage current still remained to be below 1.9 × 10^−9^ μA/μm at *V*_ds_ = 1 V and *V*_g_ = −50 V. Note that the threshold voltage (*V*_t_) shifted with increasing amplitude of *V*_ds_, because the strong drain electric field penetrated into the channel region and thinned the barrier, resulting in compromised gate-control capability, which was named by the drain-induced barrier lowing (DIBL) effect^40^. The source-drain current *I*_ds_ on/off ratio at different *V*_ds_ was summarized in Fig. 4d, showing that the on/off ratio of p-type SJFET was tuned continuously more than 10^6^ and all off-state currents were suppressed at 2.6 × 10^−8^ μA/μm. Meanwhile the on-state *I*_ds_ of SJFET in the offset geometry were not decreased in comparison with that of WSe_2_ FET with the symmetric top contact geometry, as shown in Supplementary Fig. 14.

The off-state leakage power consumption was calculated by *P*_static_ = *V*_ds_ × *I*_ds_. When *V*_ds_ = 1 V, the *P*_static_ of the n-type FET was 1.8 × 10^−5^ nW at *V*_g_ = −50 V with a high on/off ratio, although the p-type FET shows a higher *P*_static_ of 2.7 × 10^−4^ nW at *V*_g_ = 50 V. To verify the necessity of the offset contact geometry, we also measured the asymmetric FET in the top contact and bottom geometries, both of which could not simultaneously achieve the ambipolarity to unipolarity conversion (low leakage current) and a high *I*_ds_ on/off ratio (Supplementary Fig. 15). Meanwhile, we also replaced the bottom electrode with the Au film, as shown in Supplementary Fig. 16. The bottom-Au-contacted FET showed poor reconfigurability, verifying the WTe_2_ indeed played an important role in the polarity control. In addition, the effective two-terminal field-effect mobility (*μ*_eff_) for electron and hole on varied *V*_ds_ were also calculated by Eq. (7):

7 $${\mu }_{{{{{\rm{eff}}}}}}=\left(\frac{{{{{\rm{d}}}}}{I}_{{{{{\rm{ds}}}}}}}{{{{{\rm{d}}}}}{V}_{{{{{\rm{g}}}}}}}\right)\left(\frac{L}{W{C}_{{{{{\rm{i}}}}}}{V}_{{{{{\rm{ds}}}}}}}\right)$$

where *V*_g_ is the applied back gate voltage and *C*_i_ is the capacitance of the SiO_2_ dielectric layer (~11.5 nF/cm^2^). Figure 4d shows that the *μ*_eff_ of electron for the WSe_2_ FET was almost twice the *μ*_eff_ of hole for the device with the MGr contacts. Both *μ*_eff_ of electron and hole were strongly influenced by *V*_ds_ since the calculated effective *μ*_FET_ were limited by the contact barrier. To qualify the *V*_ds_-induced switching of transport polarity, we measured the barrier heights of the asymmetric SJFET at variable temperatures (Supplementary Fig. 17). The Φ_B-n_ and Φ_B-p_ of top-MGr contact were obtained from the slope of a linear fit to ln (*I*_ds_/*T*^1.5^) as a function of 1/*k*_B_*T*, by employing the 2D thermionic emission Eq. (6). Figure 4e, f show that the Φ_B-n_ at *V*_ds_ = 1 V was extracted at *V*_FB_ = 17 V to be 79 meV and the Φ_B-p_ at *V*_ds_ = −1V was extracted at *V*_FB_ = −3V to be 142 meV, although the p-branch *I*_on_ was slightly higher than the n-branch *I*_on_. The calculated results indicated that both p- and n-*I*_on_ were mainly attributed to the tunneling currents, hence the barrier width instead of height determined the on-state current density.

Figure 4g shows the reconfigurable rectifying behavior of the SJFET with different *V*_g_ in which the rectifying direction was switched by *V*_g_. The maximum rectifying ratio reached 3×10^6^ in the positive rectifying direction and 2.5 × 10^5^ in the negative rectifying direction, whereas the rectifying ratio of the all-MGr-contacted WSe_2_ Schottky diode was only about 10 at *V*_ds_ = ±1 V. To further evaluate the rectifying performance of the gate-tunable WSe_2_ Schottky junction diode, an ideality factor (*n*) was estimated at a small forward bias (here is 0.02–0.35 V) by fitting to Schottky diode Eq. (8).

8 $${I}_{{{{{\rm{ds}}}}}}={I}_{{{{{\rm{s}}}}}}\left[\exp \left(\frac{{V}_{{{{{\rm{ds}}}}}}}{n{V}_{{{{{\rm{T}}}}}}}-1\right)\right]$$

where *I*_ds_, *I*_s_, *V*_ds_, and *V*_T_ denote the drain current, reverse leakage current, drain voltage, and thermal voltage, respectively. As the gate voltage swept from positive to negative in Fig. 4h, the ideality factor *n* derived from the parameters of the fitting equation was nearly fixed on 1 with negligible variation, indicating a near-ideal diode attribute in the reconfiguration process. Figure 4i summarizes the reported ideality factors and off-state current of the 2D SJFET, indicating the high quality of the asymmetric contacted SJFET and the application potential towards lower static power dissipation.

The reconfigurable rectifying operation was based on unpinned energy level at the MGr/WSe_2_ interface and the strong carrier-injection suppression capability of WTe_2_. As shown in Supplementary Fig. 18, when *V*_g_ > 0 at *V*_ds_ > 0, the gate-electric field induced strong electron accumulation and reduced the ψ_s_ of the MGr-contacted WSe_2_. Hence the SB width was thinned to promote the electron injection from MGr through the DT (Supplementary Fig. 18b). In contrast, when *V*_g_ < 0, the width of barrier at WTe_2_/WSe_2_ interface remained constant due to the shielding effect, which reduced the off-state hole current leakage (Supplementary Fig. 18c). Reversed carrier injection process happened at *V*_ds_ < 0 (Supplementary Fig. 18d–f), only holes were allowed to be injected from the MGr side when *V*_g_ < 0. The WTe_2_ contacting strategy can also be applied to fabricate the reconfigurable MoS_2_ SJFET (Supplementary Fig. 19). The SJFET with Au/WTe_2_ contacts showed a gate-tunable rectifying characteristic with rectification ratios ranging from 1 to 10^5^. Compared to similar transport curves of Au/MGr contacted SJFET at *V*_ds_ = ±1 V (Supplementary Fig. 19d and e), the transport behavior of Au/WTe_2_ contacted device was determined by the sign of *V*_ds_, because the electron injection from WTe_2_ was inhibited, as shown in Supplementary Fig. 19c.

### Gate-tunable photo-response of the SJFET

**Fig. 5:**
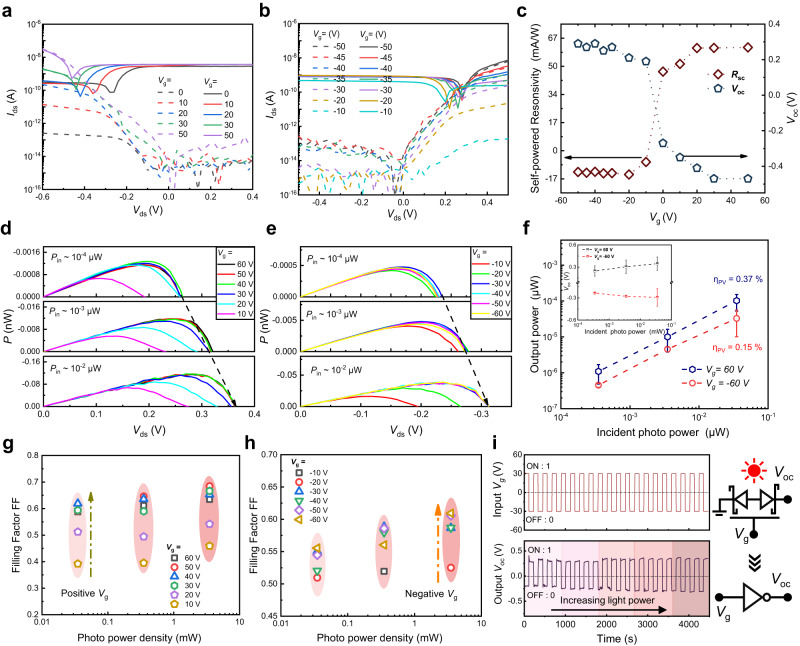
The photovoltaic performance of asymmetric contacted SJFET. **a**, **b** Gate-dependent output curves of the device under illumination (635 nm). Dashed lines were measured in the dark. **c** Gate modulation of the responsivity (*R*_sc_) at *V*_ds_ = 0 V and open-circuit voltage *V*_oc_, respectively. **d**, **e** Output electrical powers at *V*_g_ > 0 and *V*_g_ < 0 as a function of drain-source voltage, respectively. *P*_in_ is the incident-light intensity. The dashed line indicates the increasing trend of *V*_oc_ with *P*_in_. **f** Output electrical power at *V*_g_ > 0 and *V*_g_ < 0 as a function of incident-light density. The inset shows *V*_oc_ vs incident power density. the incident power density. η_PV_ is the power conversion efficiency. The standard deviations were used as error bars. **g**, **h** Fill factor (FF) at *V*_g_ > 0 and *V*_g_ < 0 as a function of the incident power density. The FF increased with the increasing amplitude of *V*_g_. **i** Logic inverter based on the gate switchable photovoltaic performance. The white light power intensity was ranged from 0.1 mW/cm^2^ to 30 mW/cm^2^.

Because the SJFET is regarded as equivalent to two back-to-back Schottky junctions at the asymmetric contact interfaces, the photo-response was tuned by both the *V*_ds_ and the *V*_g_. We used two devices to investigate the photo-response and the optical image are shown in Supplementary Fig. 10a, b. The data in Fig. 5a–c were derived from sample 2#. We first investigated the photocurrent *I*_p_ and photoresponsivity at positive and negative *I*_ds_ when *V*_g_ = 0 (Supplementary Fig. 20), which showed a nearly linear increase with the laser power intensity. More details of power-dependent photo-response are shown in Supplementary Note 4. More importantly, the SJFET also showed a potential as a self-powered photodetector due to its tunable photovoltaic performance. Supplementary Fig. 20c shows the power-dependent temporal short-circuit current *I*_sc_ at *V*_g_ = 0. The *I*_sc_ was slightly lower than the photocurrents at the same power density, but the photovoltaic response had a smaller dark current and a lower power consumption since *V*_ds_ was not required.

The photovoltaic responses of the WSe_2_ FET were further tuned by *V*_g_. Figure 5a, b shows the output curves on varying positive and negative *V*_g_ under the same laser power series (*P*_in_ = 3.5 mW/cm^2^, and a wavelength of 635 nm). With *V*_g_ positively increasing, *I*_sc_ and open-circuit *V*_oc_ gradually entered the saturated region. The negative *V*_g_ modulated behavior was similar. The gate-tunable *V*_oc_ and *I*_sc_ were summarized in Fig. 5c, showing that the *V*_oc_ was tuned from 0.29 V to −0.47 V and the self-powered responsivity *R*_sc_ was tuned from −12.7 mA/W to 61.7 mA/W. Figure 5d, e shows that regardless of the positive and negative gate bias, *I*_sc_ and output electrical power density showed an exponential increase with light power intensity, and *V*_oc_ also monotonically increased (Fig. 5f). Hence the power conversion efficiency η_PV_ calculated by η_PV_ = *P*_out_/*P*_photo_ was almost fixed at 0.37% at *V*_g_ = 60 V and 0.15% at *V*_g_ = −60 V, respectively, although the incident power increased by two orders of magnitude. Although the output electrical power density and *V*_oc_ were effectively modulated through the gate control under varying laser power density, *I*_sc_ showed a weak gate-tunable capability, in which the corresponding self-powered responsivity reached 30 mA/W at *V*_g_ > 10 V and 1 mA/W at *V*_g_ < −10 V, respectively. The filling factor (FF) qualifies how closely a photovoltaic device acts as an ideal source. Figure 5g, h show the gate-modulated FF of the SJFET at varying incident power density due to the change of *V*_oc_. As the amplitude of *V*_g_ increased, the SJFET yielded an increased FF, reaching 0.60 at *V*_g_ = −60 V and 0.68 at *V*_g_ = 60 V. Supplementary Table 1 shows a photovoltaic performance comparison among the asymmetric contacted WSe_2_ SJFET and the previously reported photovoltaic devices, which implies the high photovoltaic performance of WSe_2_ SJFET at both positive and negative *V*_g_. The reversible photovoltaic performance rendered the asymmetric SJFET to work as the self-powered logic inverter at an ambient light level, as shown in Fig. 5i and Supplementary Fig. 20d, with *V*_g_ as the input signal and *V*_oc_ as the output signal. Even at low illuminance level (*P*_in_ = 0.1 mW/cm^2^), the logic inverter still showed the obvious *V*_oc_ switch from −0.2 V to 0.28 V, which further decreased the static power dissipation in integrated circuits due to null *V*_ds_ applied on the SJFET.

## Conclusions

In conclusion, we proposed a contact-engineered SJFET with the reconfigurable polarity and low leakage current, achieved by employing the asymmetrically vdWs semimetal contacts in which the carriers were only injected from the MGr contact and the injection was suppressed at the epitaxially-grown WTe_2_ bottom contact. The asymmetrically contacted WSe_2_ SJFET in the offset geometry showed the conversion between ambipolarity and unipolarity and the alternative carrier polarity was determined by the drain bias. Meanwhile, the leakage currents were effectively suppressed to 2 × 10^−9^ μA/μm and the device showed a controllable *I*_ds_ on/off ratio with a maximum of 10^6^. The off-state leakage power consumption was reduced to 10^−5^ nW (n-type) and 10^−4^ nW (p-type) at *V*_ds_ = ± 1 V. Also, the WSe_2_ SJFET also exhibited a reversible rectifying behavior with a maximum rectifying ratio of 3 × 10^6^ and an ideality factor of 1. Advantageously from the electrically gate-tuned contact barrier, the drain-engineered SJFET exhibited a runtime reversible photovoltaic performance in which the sign of the photo-responsivity was substantially tuned and the *V*_oc_ was switched markedly between the −0.47 V and 0.29 V. Furthermore, based on the photovoltage-reversible properties of the photodiode, a logic optoelectronic device was designed to realize the switch between positive situation to negative situation by manipulating the gate voltage. This contact engineering strategy is generally applicable to other 2D materials such as the electrically gate-tunable n-type MoS_2_ Schottky diode. The modulation of carrier injection in 2D materials also provides an alternative route to reduce the logic-circuit complexity and promises innovation for the future applications of computational sensors and optical communications.

Note: during revision of this manuscript, we became aware of a related work^20^.

## Methods

One-step epitaxial growth of WTe_2._ The molten-salt-assisted thermal chemical vapor deposition (CVD) method was used to synthesize WTe_2_. A mixture of 20 mg hydrate (NH_4_)_6_Mo_7_O_24_·4H_2_O, (NH_4_)_10_W_12_O_41_·*x*H_2_O (Sigma-Aldrich) and sodium cholate (Sigma-Aldrich) in a mass ratio of 5:5:1 and the SiO_2_ substrate was placed in the middle of the heating zone, with a Te lump placed 1 cm away from the substrate. Throughout the growth process, a carrier gas mixture of H_2_/Ar at a flow rate of 10/100 sccm was utilized. The temperature of the heating zone gradually increased to 760–860 °C and held for 3–5 min. By using these two mixed hydrates as the precursor, the MoTe_2_/WTe_2_ semimetal heterostructures were epitaxially synthesized in a one-step method in which the thicker MoTe_2_ flakes were synthesized firstly, then the WTe_2_ epitaxially were grown along the edges of MoTe_2_. As the reaction time increased, the interspaces of MoTe_2_ frameworks were covered with the polycrystalline WTe_2_ to form a continuous MoTe_2_/WTe_2_ film, as shown in Supplementary Fig. 2e. More information about the sample growth is detailed in Supplementary Note 1.

Material characterization. AFM (Bruker, Dimension Icon) in the tapping mode TUNA mode were employed to measure the thickness of device, while the contact potentials of the different areas were measured via the Kelvin probe force microscopy. Micro-Raman investigation was performed using HORIBA LabRAM HR Evolution system with 532 nm laser excitation (the laser spot was ∼1 μm). The morphology and chemical composition distribution of WTe_2_/MoTe_2_ were analyzed by SEM, and XPS (Thermo Fisher Scientific, K-Alpha+). The crystal structure of 2D flakes was characterized by the TEM (FEI Tecnai F200 systems) operated at 80 kV. The TEM sample was prepared using PMMA-supported wet-transfer method.

Electrical characterization. The SJFET devices were tested in a Cascade probe station under high vacuum conditions. The electrical measurement was performed through the Keithley 4200 semiconductor characterization system. Electrical conductivity measurements were taken from 340 K to 80 K with a cooling rate of 2 K/min. The dwell time at each test temperature was 10 min. The 635 nm lasers were used for light illumination and controlled by the Thorlabs ITC 4001. The power density was 30 mW/cm^2^. During the measurements, the devices were positioned at the center of the light spot.

## Supplementary information

Supplementary Information

Peer Review File

## Data Availability

Relevant data supporting the key findings of this study are available within the article and the Supplementary Information file. All raw data generated during the current study are available from the corresponding authors upon request.

## Code availability

The code that supports the findings of this study is available from the corresponding author upon request.
