# Supplementary material for: Contact-engineered reconfigurable two-dimensional Schottky junction field-effect transistor with low leakage currents
Source: Nat Commun. 2023 Jul 17;14:4270. doi: 10.1038/s41467-023-39705-w (PMC10352327; doi:10.1038/s41467-023-39705-w)
Supplement: Supplementary file 1 — Supplementary Information [file 41467_2023_39705_MOESM1_ESM.pdf]

Supplementary Information

Contact-engineered reconfigurable two-dimensional Schottky junction field-effect transistor with low leakage currents

Yaoqiang Zhou[1], Lei Tong[1], Zefeng Chen[2], Li Tao[3], Yue Pang[1], Jian-Bin Xu[1, *]

1, Department of Electronic Engineering and Materials Science and Technology Research Center, The Chinese University of Hong Kong, Hong Kong SAR, China

2, School of Optoelectronic Science and Engineering and Collaborative Innovation Center of Suzhou Nano Science and Technology, Soochow University, Suzhou 215006, China

3, Key Lab of Advanced Optoelectronic Quantum Architecture and Measurement (Ministry of Education), School of Physics, Beijing Institute of Technology, Beijing 100081, China

*Corresponding author: jbxu@ee.cuhk.edu.hk

Inventory of Supporting Information

Supplementary Notes......1

Supplementary Note 1. Epitaxial growth and characterization of WTe_2......1

Supplementary Note 2. Electrical measurement of WTe_2 and $MoTe_2$ 2

Supplementary Note 3. Raman spectroscopic characterization of WSe_2 on different substrates......3

Supplementary Note 4. Power-dependent photo response of the SJFET 4

Supplementary Figures......5

Supplementary Figure 1. Electric characteristics of MGr-contacted WSe_2 SJFET in offset geometry. 5

Supplementary Figure 2. One-step epitaxial growth of WTe_2. 6

Supplementary Figure 3. Chemical analysis of the epitaxially-grown WTe_2......7

Supplementary Figure 4. Morphological and Raman spectroscopic characterization of epitaxially grown WTe_2 8

Supplementary Figure 5. Resistance measurement of WTe_2 by two-terminal and four-terminal

methods..9
Supplementary Figure 6. Thickness measurement of WTe_2 used in the electrical measurement....10
Supplementary Figure 7. Electrical measurements of the WTe_2 FET at varied temperatures measured by two-terminal method...11
Supplementary Figure 8. Transfer curves of the WSe_2 FET in top contact geometry....................12
Supplementary Figure 9. Carrier injection capability of the bottom MGr and WTe_2 contacts. 13
Supplementary Figure 10. Distribution of electric field and carrier density through the SJFET with different geometries simulated using the COMSOL Multiphysics package. 14
Supplementary Figure 11. Height and potential measurement of the WSe_2/WTe_2 and MGr/WTe_2. 15
Supplementary Figure 12. Surface potential measurement of WSe_2 on different substrates by Kelvin Probe Force Microscopy. 16
Supplementary Figure 13. Raman spectroscopic characterization of WSe_2 on the different substrates. 17
Supplementary Figure 14. Comparison of the on-state I_{ds} among the symmetric and asymmetric contacted WSe_2 FET...18
Supplementary Figure 15. Electrical measurement of asymmetrically contacted WSe_2 FET. 19
Supplementary Figure 16. Electrical measurement of bottom-Au-contacted WSe_2 FET...............20
Supplementary Figure 17. Barrier height calculation of asymmetrically contacted FET................21
Supplementary Figure 18. Operation mechanism of reconfigurable MGr-WSe_2-WTe_2 SJFET in offset contact geometry. 22
Supplementary Figure 19. Performance comparison between Au/WTe_2 and Au/MGr contacted MoS_2 Schottky barrier FET..23
Supplementary Figure 20. Photo-response of the asymmetrically contacted diode at V_g= 0. 24
Supplementary Table...25
Supplementary Table 1. The photovoltaic photo-response of this asymmetrically contacted WSe2 SJFET device compared to a few previously reported photovoltaic devices, implying the high performance of the present photovoltaic detector. 25
Supplementary References..26

Supplementary Notes

Supplementary Note 1. Epitaxial growth and characterization of WTe_2

The molten-salt-assisted chemical vapor deposition (CVD) method was used to synthesize $MoTe_2$ and WTe_2 by using hydrate $(NH_4)_6Mo_7O_{24}{\cdot}4H_2O$ and $(NH_4)_{10}W_{12}O_{41}{\cdot}xH_2O$ as the Mo and W sources, respectively [1]. The growth of WTe_2 was limited by the lower volatilization rate of the W source due to the high melting point of WO_3, so the WTe_2 flake usually possessed a small size at 780 °C (Supplementary Figure 2a). Reversely, at the same reaction temperature, the $MoTe_2$ showed a different morphology with a large-size ribbon-like shape because of the higher supply rate of the Mo source as shown in Supplementary Figures 2b and 2c.

By using these two mixed hydrates as the precursor, the $MoTe_2/WTe_2$ semimetal heterostructures were synthesized in a one-step method, in which the thicker $MoTe_2$ flakes were synthesized firstly and worked as the growth seeds to provide the nucleation sites and reduce the energy barrier of WTe_2 growth (Supplementary Figure 2d). Then the WTe_2 with a thin thickness was epitaxially grown along the edges of $MoTe_2$. As the reaction time increased, the interspaces of $MoTe_2$ frameworks were covered with the polycrystalline WTe_2 to form a continuous $MoTe_2/WTe_2$ film as shown in Supplementary Figure 2e.

This thickness-dependent composition ratio of Mo and W elements in epitaxially-grown WTe_2 was investigated by the energy-dispersive X-ray spectroscopy (EDS) as shown in Supplementary Figures 3a-3d. Compared to the uniformly distributed Te atoms, the Mo element was distributed in the thick seed region, while the W element was preferred to be distributed in the thin edging region. X-ray photoelectron spectroscopy (XPS) was also employed to analyze the thickness-dependent compositions of the epitaxially-grown WTe_2. The representative XPS survey spectra shown in Supplementary Figure 3e indicated that the observed peak positions are consistent with the binding energies of Mo, W, and Te elements, in which the intensity of Mo characteristic peaks decreased significantly as the thickness of the $MoTe_2/WTe_2$ layer decreased. Furthermore, the relative amount of constituent elements can be semi-quantitative analyzed by the peak intensity in XPS spectra. The atom ratio of W and Mo in WTe_2 and $MoTe_2$ samples can be estimated by comparing the intensities of the XPS signal by using the Te peak as the reference. The Mo spectra in Supplementary Figure 3f show that as the thickness decreases, the signal from Mo becomes weaker, whereas the atom ratio

of W/Mo x is enhanced. The corresponding stoichiometric ratio of W element x was calculated to be 0, 0.52, and 0.96, respectively as shown in Supplementary Figure 3g, where the x and the morphologies were highly correlated: thin $MoTe_2/WTe_2$ tended to possess higher W atomic concentration.

We used high-resolution transmission electron microscopy (HRTEM) and Raman spectrometer to probe the quality of WTe_2. Supplementary Figures 2f-2g show the TEM image of a ribbon-like WTe_2. Supplementary Figure 4b shows the line scan of Raman spectra across the $MoTe_2$ /WTe_2 heterostructure and the characteristic peaks are marked with corresponding vibration modes: the A_1^9 (214 cm^{-1}) mode and A_g (268 cm^{-1}) modes are denoted as the fingerprint vibration modes in WTe_2 and $MoTe_2$, respectively. When the laser was irradiated on the interface of $MoTe_2$ and WTe_2, the Raman signals from both parts were collected, which induced the gradual transition in the line-scanning Raman spectra [2]. The phase-contrast imaging in atomic force microscopy (AFM) measurement in Supplementary Figure 4c showed $MoTe_2/WTe_2$ interface clearly due to the variation in tip-sample dissipation over the $MoTe_2$ and WTe_2 regions, which indicated this sharp interface at the position of the step edge. Supplementary Figures 4d and 4e show the polarized Raman spectra measured in an incident laser rotation configuration. A vertical direction (white arrow in Supplementary Figure 4f) is defined in zero degrees. Raman modes (A_1^9) of WTe_2 and (A_g) of $MoTe_2$ are linearly polarized and show a consistent two-fold symmetric intensity curve [3, 4], indicating the high quality of the epitaxially-grown sample. These characterization results indicated the seamless and reliable connection between $MoTe_2$ and WTe_2, which means the $MoTe_2/WTe_2$ heterojunctions can work as ultrathin van der Waals contacts to build 2D Schottky junctions.

Supplementary Note 2. Electrical measurement of WTe_2 and $MoTe_2$

Two-terminal Resistance Measurements

As a common resistance measurement method for 2D devices as shown in Supplementary Figure 5a, the two-terminal resistance especially for low channel resistance is not accurate because the contact resistance (R_c) cannot be excluded. The test current I caused a significant voltage-drop across the R_c, which results in a larger measured voltage (V_m) than the true voltage (V_R). Hence the measured resistance by two-terminal method is usually overestimated due to dominant R_c.

Four-terminal Resistance Measurements

Due to the limitations of the two-wire method, the four-terminal (Kelvin) method was used for low resistance measurements that reduced the effect of contact resistance as shown in Supplementary Figure 5b. With this configuration, the test current *I* transported through the WTe_2 resistance (*R*) via 1 and 4 electrodes, while the voltage V_m across the WTe_2 was measured through 2 and 3 electrodes. Since only a negligible current may flow through the 2 and 3 electrodes, the V_m is an accurate estimation of the true voltage across the WTe_2. As a result, the contact resistance was eliminated by using the four-terminal method.

Supplementary Note 3. Raman spectroscopic characterization of WSe_2 on different substrates

We used the Raman spectra as indirect evidence to investigate the interaction between the WSe_2 and bottom contacts. Supplementary Figures 13a and 13b show the Raman spectra of the WSe_2 on the different substrates using 532 nm laser excitation, in which one prominent peak with a small shoulder appears at around 250 cm^{-1}, and two characteristic peaks (E_{2g} and A_{1g}) are expected [5]. Supplementary Figures 13c and 13d depict the Raman spectra of WSe_2 on Au film and WTe_2, by using the WSe_2 on SiO_2 as the reference. All the peaks were indexed by employing multi-peak Lorentzian fitting. The E_{2g} and A_{1g} peaks at 246.2 cm^{-1} and 256 cm^{-1} were distinguished. The E_{2g} and A_{1g} modes correspond to the in-plane and out-of-plane vibrational modes. The intensity of the characteristic peak (E_{2g}) of WSe_2 on Au film was clearly decreased and the full width at half maximum (FWHW) also changed in comparison to that of WSe_2 on SiO_2 substrate. Although the reflectance of Au film was higher, the Raman intensity (E_{2g}) of WSe_2 on WTe_2 showed a samller decrease. More importantly, the A_{1g} mode is the out-of-plane vibrational mode involving Se atoms, which is easy to be affected by the electrostatic environment changes [6]. Our results showed that the full width at half maximum (FWHM) of the A_{1g} peak was enlarged as the surface potential of WSe_2 increased (Supplementary Figures 12e-12f), indicating the stronger hole transfer doping effect of Au film according to previous reports.

Due to gap-state induced Fermi level pinning (Supplementary Figure 14a), the bottom-Au contact only showed a hole injection capability as indicated by the transfer curves of symmetric Au-WSe_2 in the bottom contact geometry (Supplementary Figure 14b), making the MGr-WSe_2-Au SJFET in

offset contact geometry exhibiting a poor reconfigurability and leakage repression capability compared to WTe_2-bottom-contacted SJFET as shown in Supplementary Figures 14c-14d. Hence, although the Au film and WTe_2 showed a similar work function (< 60 meV), the WSe_2 still suffered more severe interlayer interaction when contacted with Au film.

Supplementary Note 4. Power-dependent photo response of the SJFET

Supplementary Figure 19a shows the light-power-dependent drain current I_{ds}-gate voltage V_g curves of SJFET at $V_g = 0$. Due to the large Schottky barrier height at both WTe_2 and MGr sides, the device showed a small dark current I_{dark} on both forward and reversed V_{ds}. Supplementary Figure 19b shows that the photocurrent I_p and photoresponsivity at V_{ds} = -0.8 and 0.2 V were approximately linearly increased with the laser power intensity. The photoresponsivity R is defined as Equation (1):

$$R=\frac{I_{ph}}{P_{in}A} \tag{1}$$

where I_{ph} is the photocurrent, P_{in} is the incident laser power density, and A is the junction area of the asymmetrically contacted WSe_2 SJFET. As a WSe_2 photoconductor, a high responsivity of 260 mA/W at V_{ds}= -0.8 V was obtained under the lowest light intensity (10^{-4} mW/cm^2) and the responsivity at V_{ds}= 0.2 V only reached 40 mA/W.

Supplementary Figures

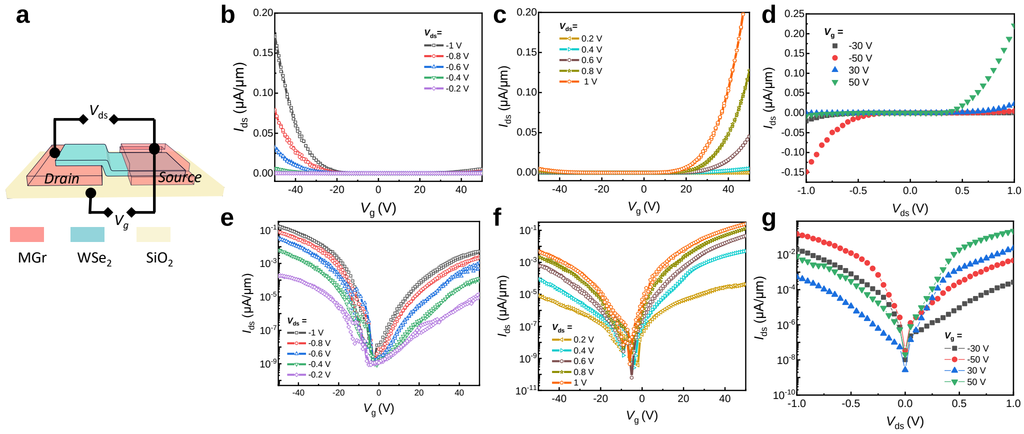

Supplementary Figure 1. Electric characteristics of MGr-contacted WSe_2 SJFET in offset geometry. a) Schematic of MGr-offset-contacted WSe_2 SJFET. b) Transfer curves of the MGr-offset-contacted WSe_2 SJFET when $V_{ds} < 0$. c) Transfer curves of the MGr-offset-contacted WSe_2 SJFET when $V_{ds} > 0$. d) Output curves of the MGr-offset-contacted WSe_2 SJFET at varying V_g. e) and f) Transfer curves of the MGr-offset-contacted WSe_2 SJFET when $V_{ds} < 0$ and $V_{ds} > 0$. g) Output curves of the MGr-offset-contacted WSe_2 SJFET at various V_g.

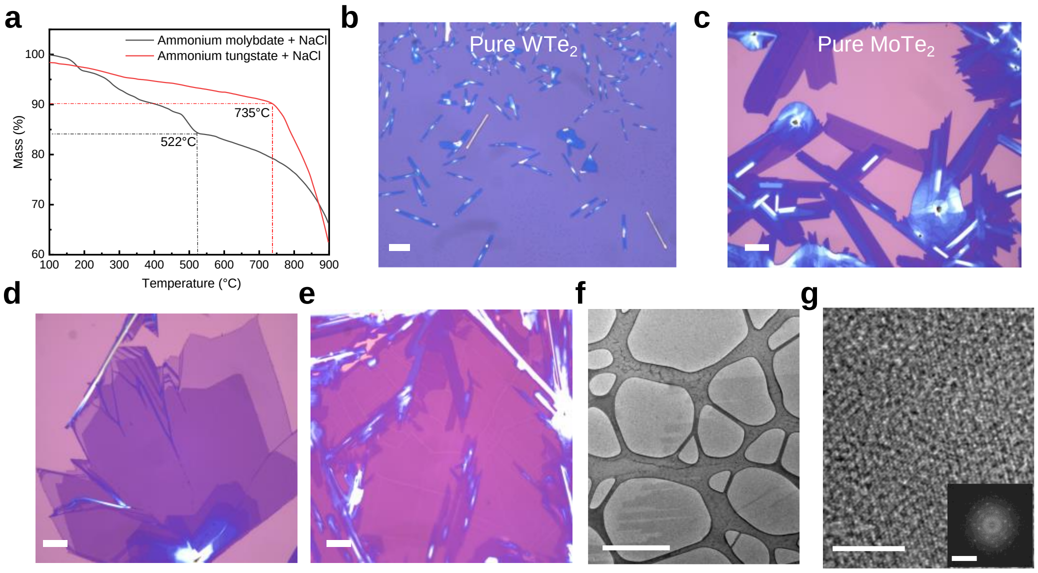

Supplementary Figure 2. One-step epitaxial growth of WTe_2. a) Thermogravimetric analysis of the W and Mo sources in the nitrogen atmosphere. b) Optical image of WTe_2 prepared only by using the W source at a growth temperature of 780 °C. Scale bar: 50 μm. c) Optical image of $MoTe_2$ prepared only by employing the Mo source at a growth temperature of 780 °C. Scale bar: 20 μm. d) Optical image of epitaxially-grown WTe_2 with a growth time of 3 min. Scale bar: 20 μm. e) Photograph of WTe_2 film with a growth time of 8 min. Scale bar: 20 μm. f) TEM micrograph of the ribbon-like WTe_2. Scale bar: 50 nm. g) High-resolution TEM micrograph of epitaxially grown WTe_2. Scale bar: 5 nm. Inset shows the corresponding FFT pattern. Scale bar: 1/5 nm.

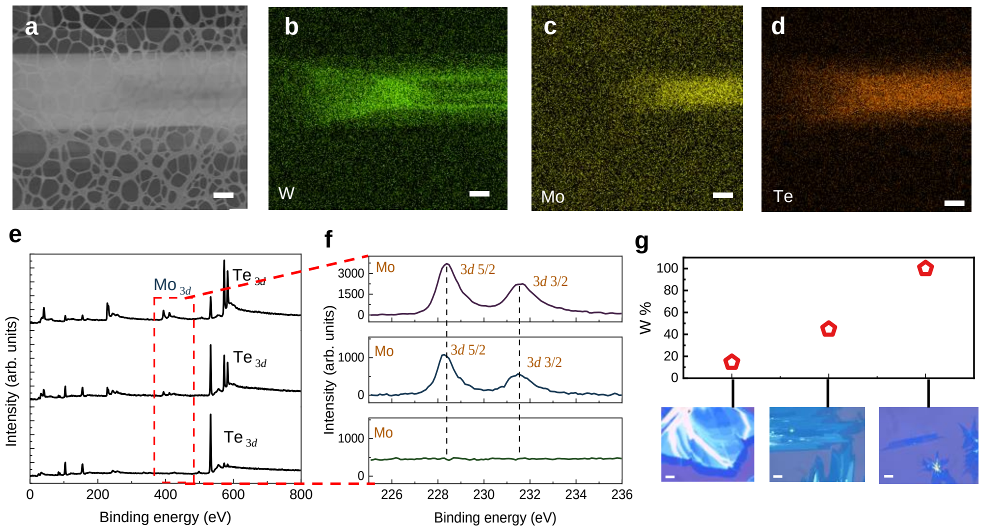

Supplementary Figure 3. Chemical analysis of the epitaxially-grown WTe_2. a) SEM micrograph of epitaxially-grown WTe_2. b)-d) EDS mapping image corresponding to the W (b), Mo (c), and Te (d) elements. Scale bar: 1 μm. e) XPS global spectra of the sample with decreased thickness. The optical images of the samples are shown in (g). f) Mo spectra of samples with decreased thickness. The characteristic peaks of Mo disappeared in the thin WTe_2 sample. g) The relationship between the Mo concentration and morphology of samples. The stoichiometric ratio of Mo and W atom were calculated based on the characteristic peak area of Te as the reference. Scale bar: 20 μm.

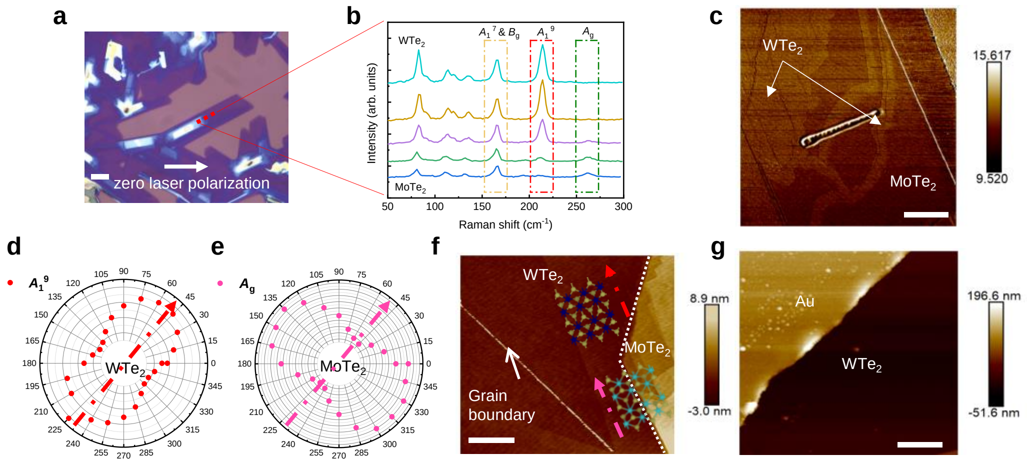

Supplementary Figure 4. Morphological and Raman spectroscopic characterization of epitaxially grown WTe_2. a) Optical image of the epitaxially-grown WTe_2. Scale bar: 20 µm. The white arrow represents the direction of zero-incident-laser polarization in Raman spectroscopy measurement. b) Raman spectra measured along the epitaxial growth direction. c) Phase contrast image of the interface of epitaxially-grown WTe_2 measured by tapping mode of AFM. Scale bar: 4 µm. d)-e) Polarized Raman spectra at the characteristic peaks of A_1^9 and A_g, measured with incident laser rotation configuration. f) Morphology of epitaxial WTe_2 measured by AFM. Scale bar: 4 µm. The inset shows the top views of lattice structures of monolayer T_d phase WTe_2 and $MoTe_2$. Brown spheres: tellurium atoms. Blue spheres: W atoms. Green spheres: Mo atoms. The dashed line indicates the direction of the *a*-axis of the WTe_2 and $MoTe_2$ crystals. g) Height image of Au film and CVD-grown $W(Mo)Te_2$. Scale bar: 2 µm.

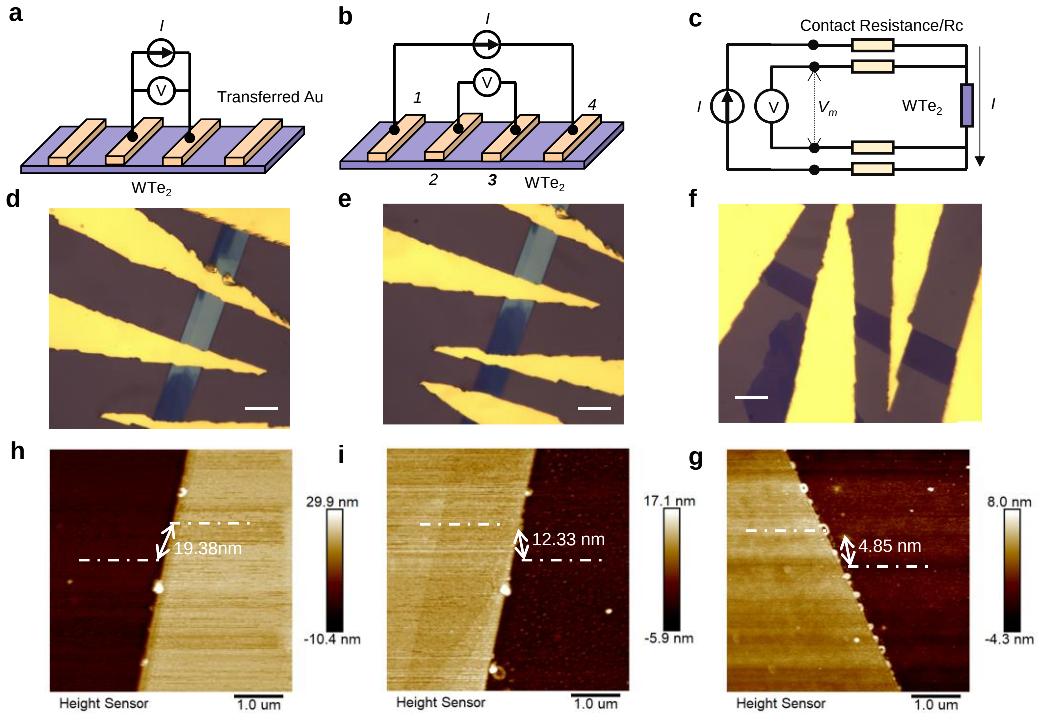

Supplementary Figure 5. Resistance measurement of WTe_2 by two-terminal and four-terminal methods. a) Schematic of two-terminal electrical measurement. b) Schematic of four-terminal electrical measurement. The transferred Au films were used as electrodes. c) Schematic of circuits of four-terminal resistance measurement. V_m is the measured voltage. d)-f) Optical images of the measured WTe_2 sample with different thicknesses. Scale bar: 10 μm. h)-g) AFM height images of WTe_2 samples with varying thicknesses used in the electrical measurement.

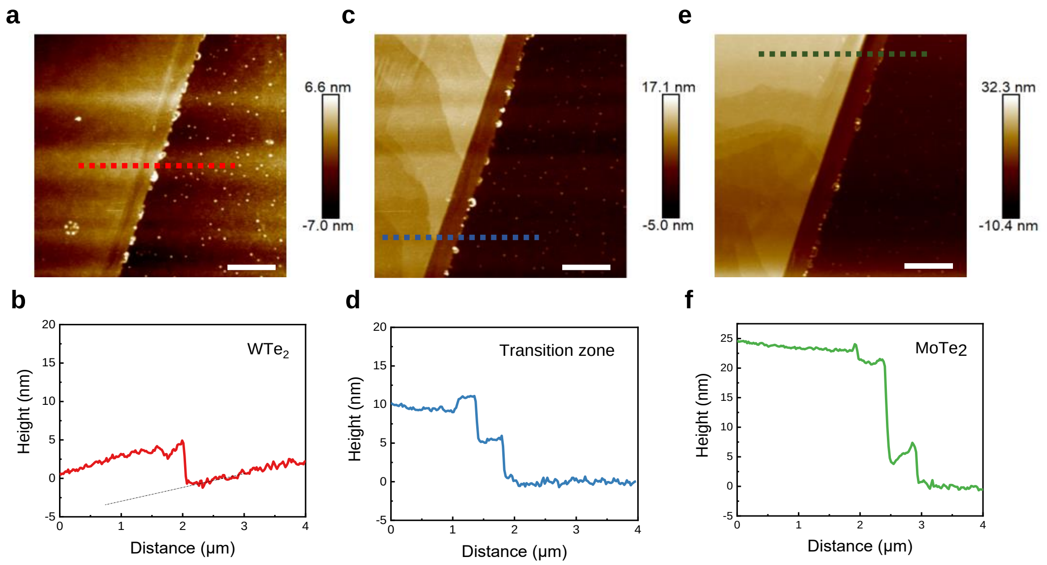

Supplementary Figure 6. Thickness measurement of WTe_2 used in the electrical measurement. a)-b) Thin WTe_2 region. Scale bar: 1 μm. c)-d) Thickness variation in the junction region. Scale bar: 1 μm. e)-f) Thick $MoTe_2$ region.

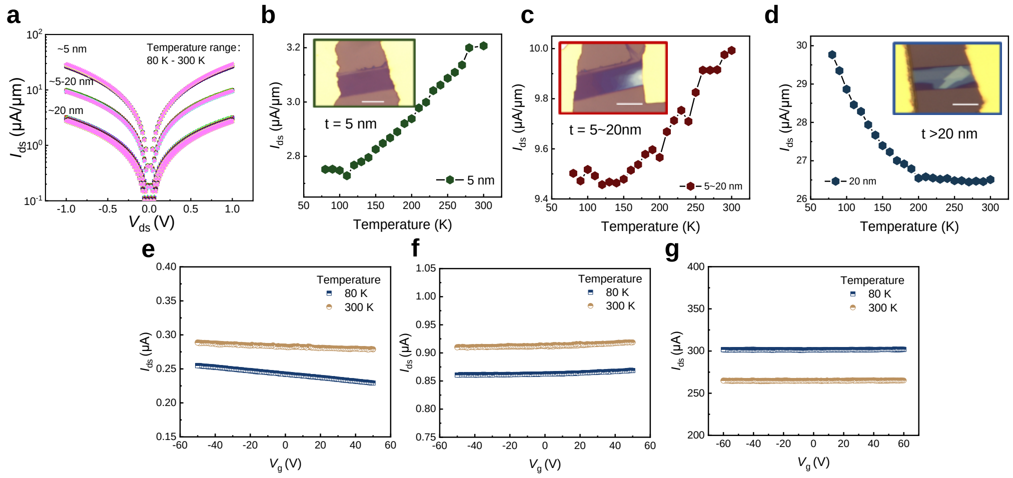

Supplementary Figure 7. Electrical measurements of the WTe_2 FET at varied temperatures measured by two-terminal method. a) I_d-V_d curves of WTe_2 FET at varying temperatures ranging from 300 K to 80 K. b)-d) Current density I_d as a function of sample temperatures, showing the thickness-dependent semiconductor-to-metal transition. Insets show photographs of epitaxially-grown WTe_2 with increasing thicknesses. Scale bar: 10 μm. e)-g) Transfer curves of WTe_2 FETs with thicknesses of 5 nm, 5~20 nm, and 20 nm at 300 K and 80 K, respectively.

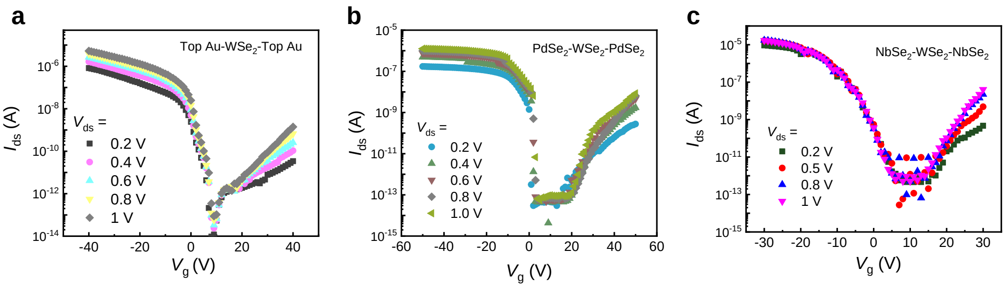

Supplementary Figure 8. Transfer curves of the WSe_2 FET in top contact geometry. a) Transfer curves of the Au-contacted FET. b) Transfer curves of the $PdSe_2$-contacted FET. c) Transfer curves of the $NbSe_2$-contacted FET.

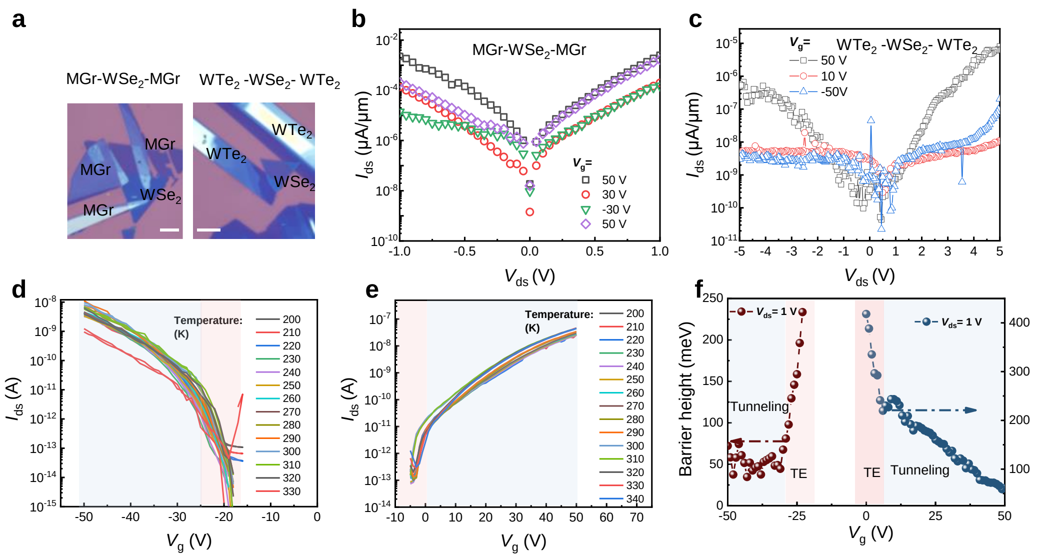

Supplementary Figure 9. Carrier injection capability of the bottom MGr and WTe_2 contacts. a) Optical images of the WSe_2 FET in bottom contact geometry. Scale bar: 10 μm. b) I_{ds}-V_{ds} curves of the MGr-contacted WSe_2 FET at varied V_g. c) I_{ds}-V_{ds} curves of the WTe_2-contacted WSe_2 FET at various V_g. The asymmetric I_{ds}-V_{ds} curves were induced by the different contact lengths at the source and drain sides. d) Transfer curves of MGr-bottom-contacted WSe_2 FET at V_{ds} = 1 V and measured at varied temperatures. e) Transfer curves of MGr-bottom-contacted WSe_2 FET at V_{ds} = -1 V and measured at varied temperatures. The blue region indicates the tunneling-dominating region and the pink region indicates the thermionic-emission-(TE)-dominating region. f) Barrier height at bottom-MGr/WSe_2 interface depending on the V_g.

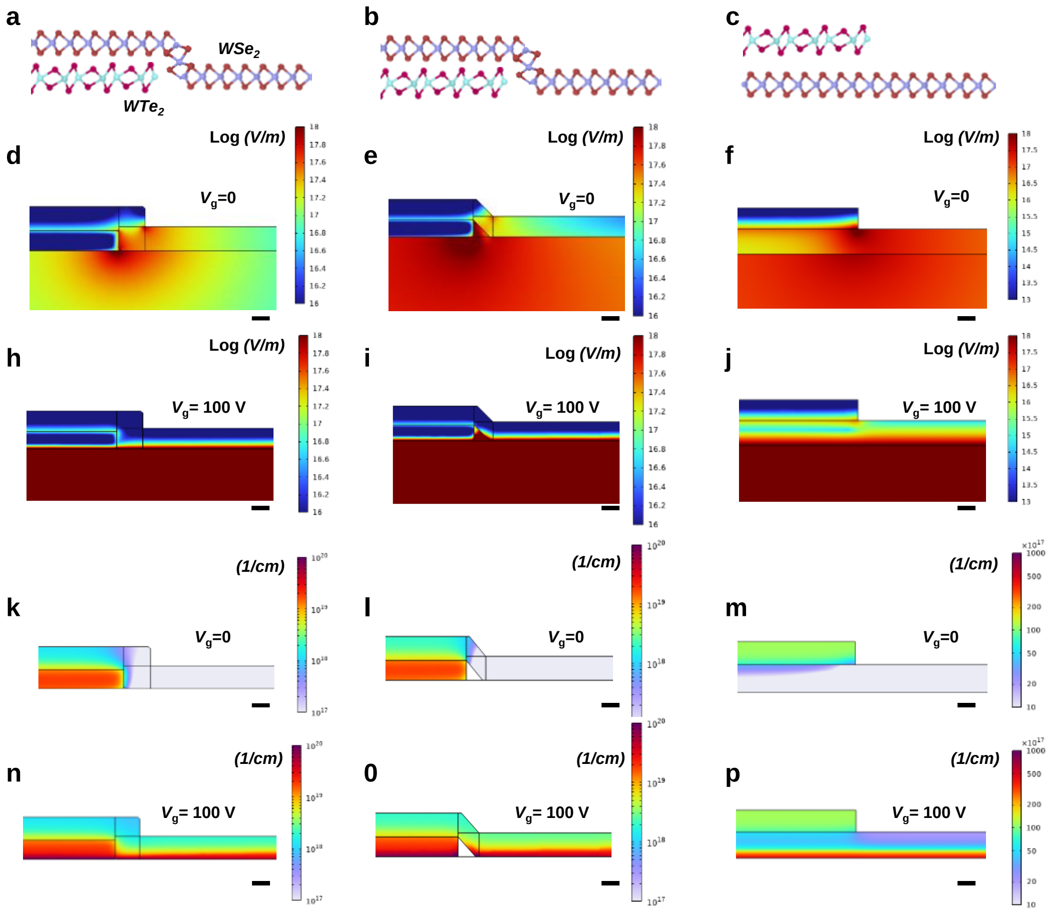

Supplementary Figure 10. Distribution of electric field and carrier density through the SJFET with different geometries simulated using the COMSOL Multiphysics package. a)-c) Schematics of the bottom and top contact geometries. d)-f) Distribution of electric field through the WSe_2 with bottom, bottom (airgap), and top WTe_2 contacts, respectively, at V_g= 0. h)-j) Distribution of electric field through the WSe_2 with bottom, bottom (airgap), and top WTe_2 contacts, respectively, at V_g= 100 V. k)-m) Distribution of electron density through the WSe_2 with bottom, bottom (airgap), and top WTe_2 contacts, respectively, at V_g= 0 V. n)-p) Distribution of electron density through the WSe_2 with bottom, bottom (airgap), and top WTe_2 contacts, respectively, at V_g= 100 V. Scale bar: 5 nm. The thicknesses of WSe_2 and WTe_2 are 8 nm and 5 nm, respectively. Due to the shielding effect of bottom contact, the electric field and carrier density of the WSe_2 upper the bottom contact was hard to be modulated by the V_g.

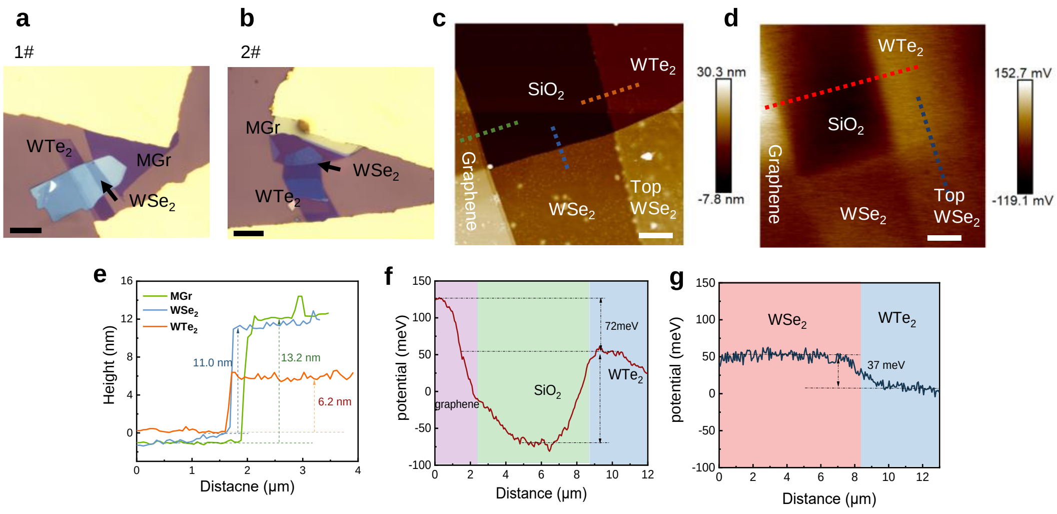

Supplementary Figure 11. Height and potential measurement of the WSe_2/WTe_2 and MGr/WTe_2. a)-b) Optical images of the WSe_2 FET in offset contact geometry. Scale bar: 10 μm. All data in Part IV electrical measurement of SJFET were measured by using sample 1#. For the Part V photo response, only the data in Figures 5a-5c were derived from sample 2#. c) AFM height image of the $MGr/MoS_2/WTe_2$ junction. d) Potential image of the $MGr/MoS_2/WTe_2$ junction. Scale bar: 3 μm. e) AFM height profiles of the MGr, WSe_2, and WTe_2 layers. f) Potential profiles across MGr, SiO_2, and WTe_2 regions along the red dash line. g) Potential profiles of WSe_2 across WTe_2 and on SiO_2 region along the blue dash line in the potential image.

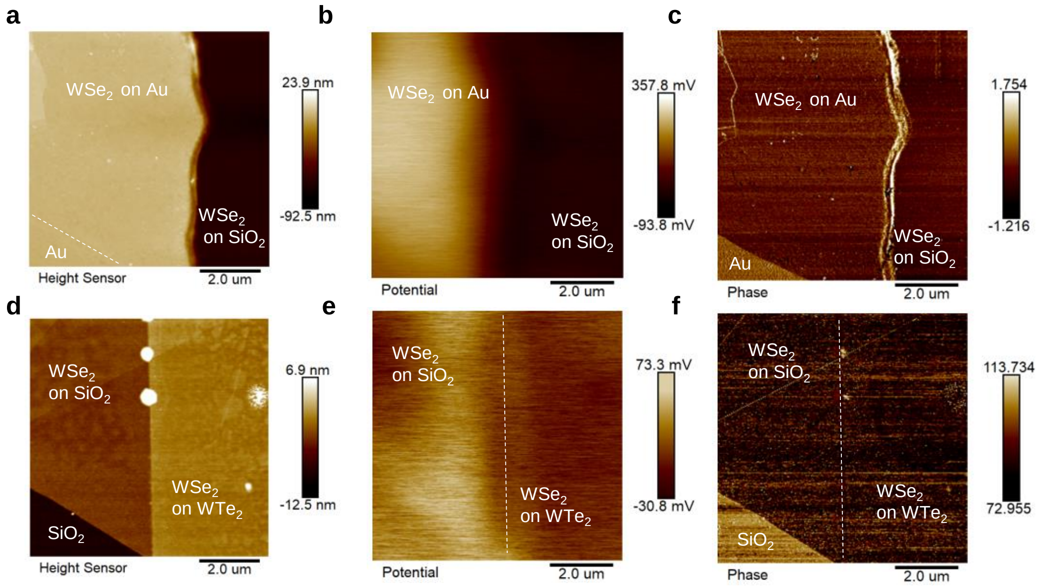

Supplementary Figure 12. Surface potential measurement of WSe_2 on different substrates by Kelvin Probe Force Microscopy. a) AFM height image of WSe_2 on the Au film and the SiO_2 substrate. b) Potential image of WSe_2 on the Au film and the SiO_2 substrate. c) Phase contrast of WSe_2 on the Au film and the SiO_2 substrate. d) AFM height image of WSe_2 on the WTe_2 flake and the SiO_2 substrate. e) Potential image of WSe_2 on the WTe_2 flake and the SiO_2 substrate. f) AFM phase contrast of WSe_2 on the WTe_2 flake and the SiO_2 substrate.

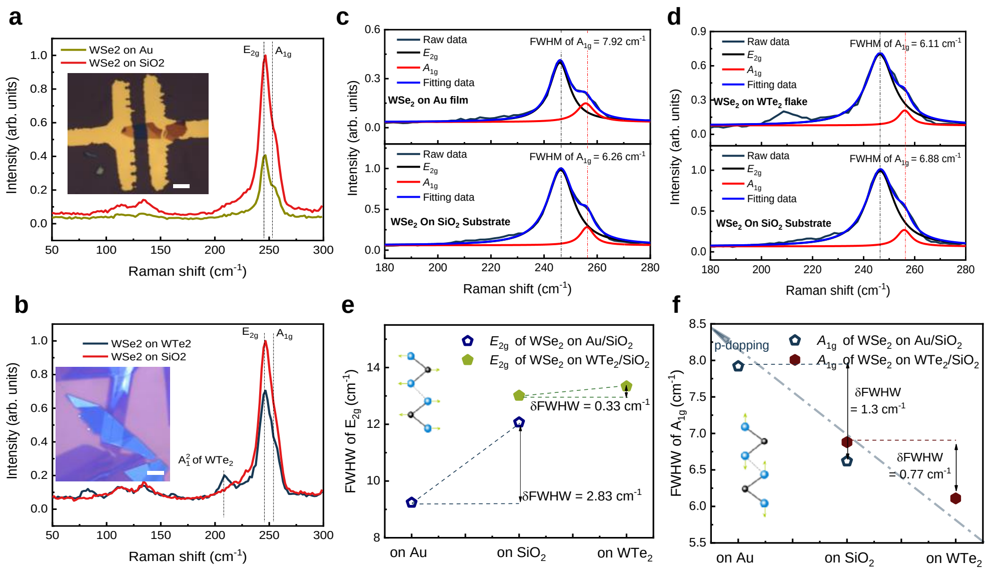

Supplementary Figure 13. Raman spectroscopic characterization of WSe_2 on the different substrates. a) Raman spectra of WSe_2 on the Au film and the SiO_2 substrate. The inset shows the optical image of the Au/WSe_2 junction. Scale bar: 10 μm. b) Raman spectra of the WSe_2 on the WTe_2 flake and the SiO_2 substrate. The inset shows the optical image of the WTe_2/WSe_2 junction. Scale bar: 10 μm. c)-d) Raman peaks and the corresponding Lorenz fittings of WSe_2 on the Au film, WTe_2 flake, and the SiO_2 substrate. e)-f) FWHW of E_{2g} and A_{1g} characteristic peaks of WSe_2 on the Au film, WTe_2 flake, and the SiO_2 substrate as the reference. Insets show the corresponding vibration modes of WSe_2. Blue spheres: selenium atom atoms. Black spheres: tungten W atoms.

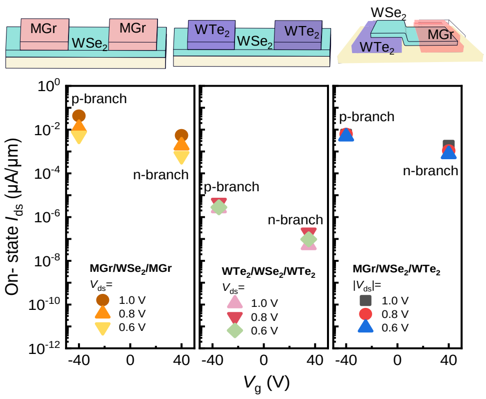

Supplementary Figure 14. Comparison of the on-state I_{ds} among the symmetric and asymmetric contacted WSe_2 FET.

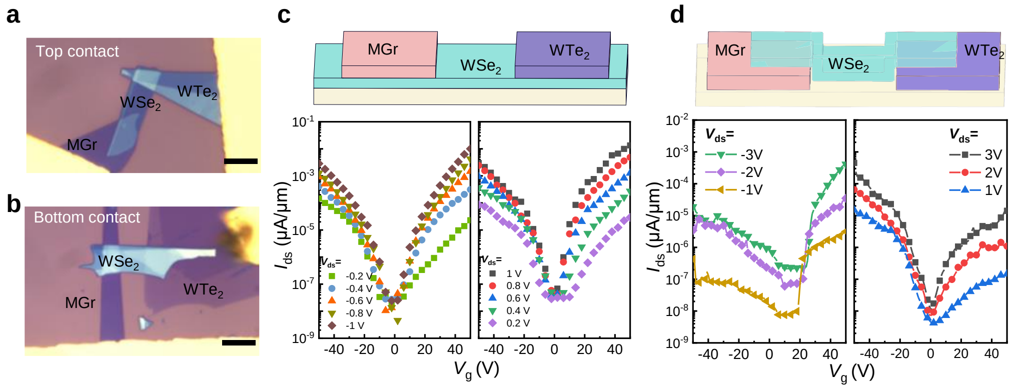

Supplementary Figure 15. Electrical measurement of asymmetrically contacted WSe_2 FET. a) Optical image of the MGr/WSe_2/exfoliated-WTe_2 FET in top contact geometry. Scale bar: 10 μm. b) Optical image of the MGr/WSe_2/ WTe_2 FET in bottom contact geometry. Scale bar: 10 μm. c) Schematic of the MGr/WSe_2/exfoliated-WTe_2 FET in top contact geometry and the transfer curves. d) Schematic of the MGr/WSe_2/ WTe_2 FET in bottom contact geometry and the transfer curves.

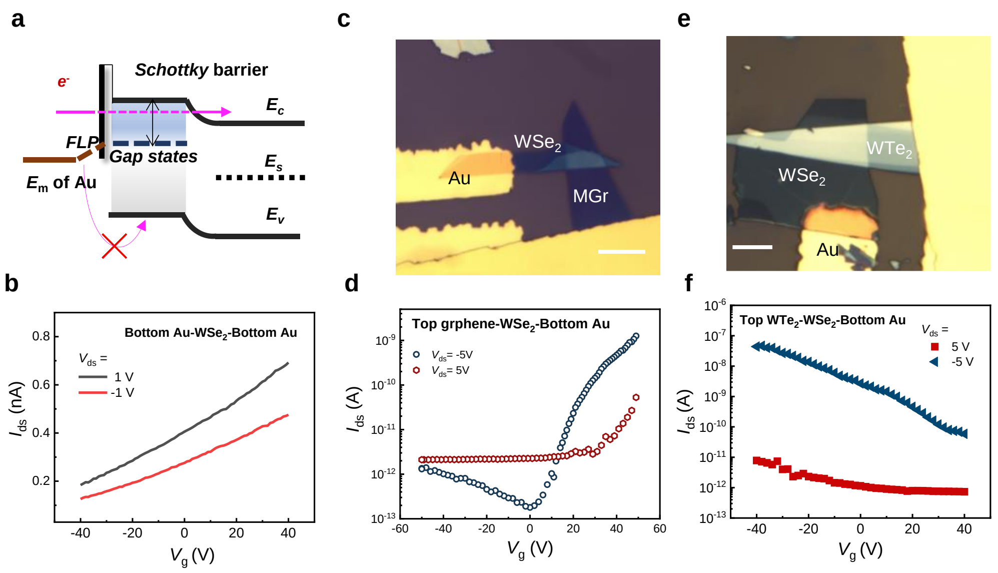

Supplementary Figure 16. Electrical measurement of bottom-Au-contacted WSe_2 FET. a) Schematic band diagram of bottom Au-WSe_2 Schottky junction. b) Transfer curves of bottom-Au-contacted WSe_2 FET. The transfer curves showed the n-type transport characteristics. c) Optical image of the bottom-Au/WSe_2/top-MGr FET. Scale bar: 10 μm. d) Transfer curves of bottom-Au/WSe_2/top-MGr FET. The shapes of transfer curves were tuned by the V_{ds}. e) Optical image of the bottom-Au/WSe_2/top-exfoliated-WTe_2 FET. Scale bar: 10 μm. f) Transfer curves of bottom-Au/WSe_2/top-exfoliated-WTe_2 FET.

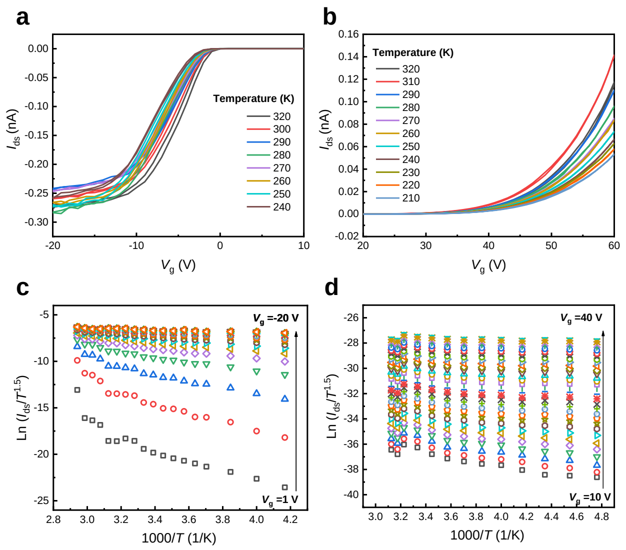

Supplementary Figure 17. Barrier height calculation of asymmetrically contacted FET. a) Transport curves of the device at varying temperatures (V_{ds} = -1 V). b) Transport curves of the device at different temperatures (V_{ds} = 1 V). c)-d) The ln ($I_{ds}/T^{1.5}$) as a function of $1/k_BT$ at V_{ds} = -1 V (c) and V_{ds} = 1 V (d).

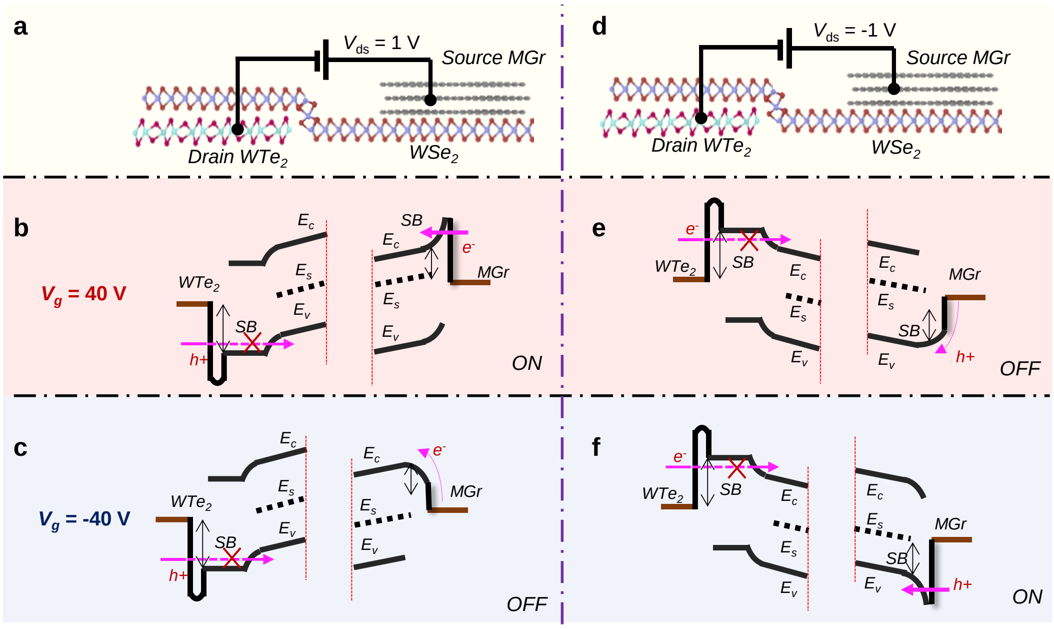

Supplementary Figure 18. Operation mechanism of reconfigurable MGr-WSe_2-WTe_2 SJFET in offset contact geometry. a) Schematic of the n-type SJFET (V_{ds} = 1 V). b) Schematic energy-band diagram of the n-type SJFET at V_g = 40 V (on-state). c) Schematic energy-band diagram of the n-type SJFET at V_g = -40 V (off-state). d) Schematic of the p-type SJFET (V_{ds} = -1 V). e) Schematic energy-band diagram of the p-type SJFET at V_g = 40 V (off-state). f) Schematic energy-band diagram of the p-type SJFET at V_g = -40 V (on-state). E_c, E_v, and E_s represent the energy of the conduction band, valence band, and Fermi level in WSe_2. SB represents the Schottky barrier.

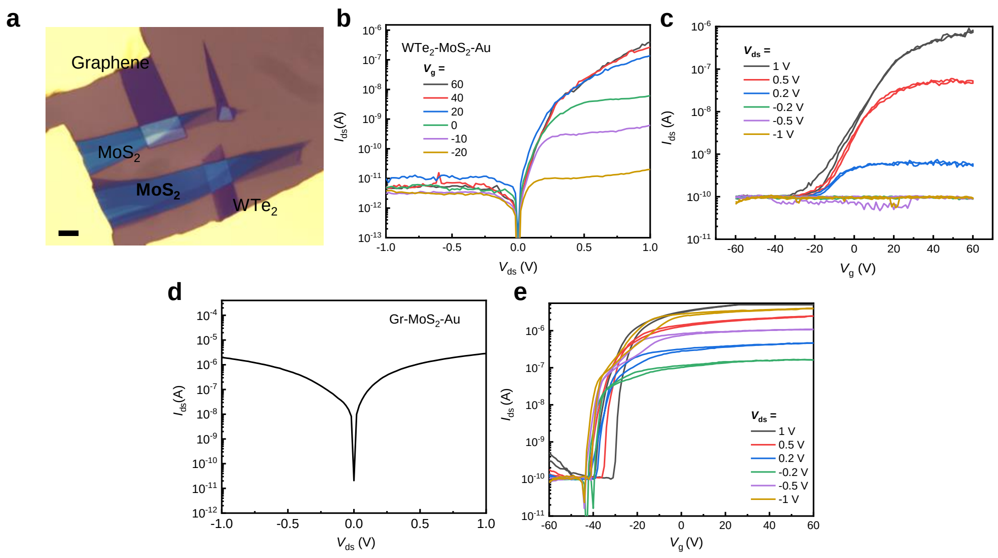

Supplementary Figure 19. Performance comparison between Au/WTe_2 and Au/MGr contacted MoS_2 Schottky barrier FET. a) Optical image of the Au/WTe_2 and Au/MGr-contacted MoS_2 Schottky barrier FET. Scale bar: 10 μm. b) Gate-dependent rectifying behavior of the Au/WTe_2 contacted diode. c) V_{ds}-dependent transfer curves of the Au/WTe_2 contacted Schottky barrier FET. Only the Schottky barrier FET with $V_{ds} > 0$ showed the n-type transport polarity. d) Symmetric I_d-V_d curve of the Au/MGr contacted MoS_2 Schottky barrier FET at $V_g = 0$. e) Transfer curves of the Au/WTe_2 contacted Schottky barrier FET with positive and negative V_{ds}.

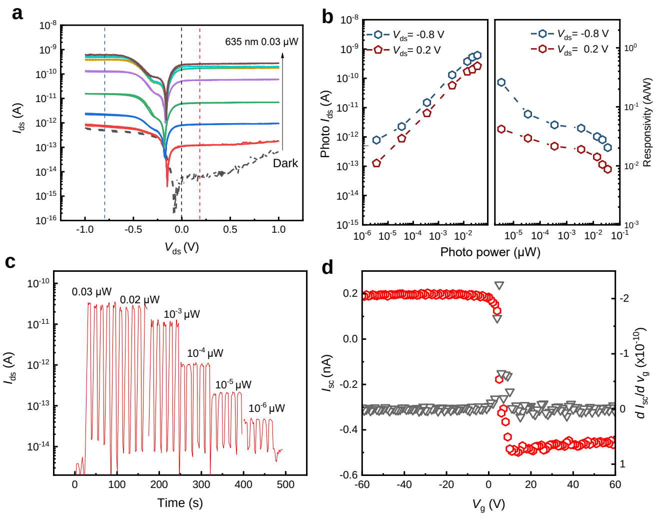

Supplementary Figure 20. Photo-response of the asymmetrically contacted diode at V_g= 0. a) Output curves of the device in the dark and under 532 nm light irradiation with various laser power densities. b) Power-dependent photoresponsivity and I_{light} /I_{dark} ratio. c) Temporal power-dependent short-circuit current I_{sc} at V_g = 0. d) Gate switchable short-circuit current I_{sc} with an incident optical power of 3.5mW/cm^2.

Supplementary Table

Supplementary Table 1. The photovoltaic photo-response of this asymmetrically contacted WSe2 SJFET device compared to a few previously reported photovoltaic devices, implying the high performance of the present photovoltaic detector.

Material & structure	Responsivity (mA/W)	PCE (%)	Maximum V_{oc} (V)	Fill Factor	Reference
WSe_2/MoS_2	120	-	0.41	-	[7]
MoS_2 PN junction	30	0.4	0.6	0.58	[8]
MoS2 PN junction	15	-	0.65	-	[9]
$MoTe_2/MoS_2$	322	-	0.32	-	[10]
Dual-gate WSe_2	16	0.5	0.86	0.5	[11]
MoS_2/BP	400	0.3	0.31	0.5	[12]
Dual-gate WSe_2	0.7	0.14	0.7	-	[13]
Ag-MoS_2-Pt	16	-	0.76	-	[14]
$MoTe_2/MoS_2$/Ti	88	-	0.2	-	[15]
Au-WSe_2-In	197	0.63	0.18~-0.2	-	[16]
Semi-gate WSe_2	170	5.62	0.62	0.67	[17]
MGr/WSe_2/WTe_2	260	0.37	0.29~-0.47	0.68	This work

Supplementary References

1. Chen, K. *et al.* A Simple Method for Synthesis of High-Quality Millimeter-Scale 1T' Transition-Metal Telluride and Near-Field Nanooptical Properties. *Adv. Mater.* **29**, (2017).
2. Choi, Y.-B. *et al.* Evidence of higher-order topology in multilayer WTe_2 from Josephson coupling through anisotropic hinge states. *Nat. Mater.* **19**, 974–979 (2020).
3. Ding, Y. *et al.* Raman Tensor of Layered Td-WTe_2. *J. Phys. Chem. C* **124**, 16596–16603 (2020).
4. Chen, K. *et al.* Lateral Built-In Potential of Monolayer MoS_2–WS_2 In-Plane Heterostructures by a Shortcut Growth Strategy. *Adv. Mater.* **27**, 6431–6437 (2015).
5. Zhao, W. *et al.* Lattice dynamics in mono- and few-layer sheets of WS_2 and WSe_2. *Nanoscale* **5**, 9677–9683 (2013).
6. Buscema, M., Steele, G. A., van derZant, H. S. J. &Castellanos-Gomez, A. The effect of the substrate on the Raman and photoluminescence emission of single-layer MoS_2. *Nano Res.* **7**, 561–571 (2014).
7. Lee, C.-H. *et al.* Atomically thin p–n junctions with van der Waals heterointerfaces. *Nat. Nanotechnol.* **9**, 676–681 (2014).
8. Lv, L. *et al.* Reconfigurable two-dimensional optoelectronic devices enabled by local ferroelectric polarization. *Nat. Commun.* **10**, 3331 (2019).
9. Pezeshki, A., Shokouh, S. H. H., Nazari, T., Oh, K. &Im, S. Electric and Photovoltaic Behavior of a Few-Layer α-$MoTe_2$/MoS_2 Dichalcogenide Heterojunction. *Adv. Mater.* **28**, 3216–3222 (2016).
10. Pospischil, A., Furchi, M. M. &Mueller, T. Solar-energy conversion and light emission in an atomic monolayer p–n diode. *Nat. Nanotechnol.* **9**, 257–261 (2014).
11. Zhang, X. *et al.* Self-Healing Originated van der Waals Homojunctions with Strong Interlayer Coupling for High-Performance Photodiodes. *ACS Nano* **13**, 3280–3291 (2019).
12. Deng, Y. *et al.* Black Phosphorus–Monolayer MoS_2 van der Waals Heterojunction p–n Diode. *ACS Nano* **8**, 8292–8299 (2014).
13. Groenendijk, D. J. *et al.* Photovoltaic and Photothermoelectric Effect in a Double-Gated WSe_2 Device. *Nano Lett.* **14**, 5846–5852 (2014).
14. Liu, Y. *et al.* Approaching the Schottky–Mott limit in van der Waals metal–semiconductor

junctions. *Nature* **557**, 696–700 (2018).

15. Zhang, X. *et al.* Near-ideal van der Waals rectifiers based on all-two-dimensional Schottky junctions. *Nat. Commun.* **12**, 1522 (2021).

16. Du, J. *et al.* Gate-Controlled Polarity-Reversible Photodiodes with Ambipolar 2D Semiconductors. *Adv. Funct. Mater.* **31**, 2007559 (2021).

17. Jin, T. *et al.* Two-dimensional reconfigurable electronics enabled by asymmetric floating gate. *Nano Res.* **15**, 4439–4447 (2022).
